# Process mapping the One Health response to a rabies outbreak in the Philippines

**DOI:** 10.1136/bmjgh-2025-020482

**Published:** 2026-04-02

**Authors:** Anna Formstone, Mary Elizabeth G Miranda, Van Denn Domingo Cruz, Duane Raphael O Manzanilla, Eleanor M Rees, Mirava Yuson, Jobin Maestro, Shynie Vee M Telmo, Daria L Manalo, Brandy Alabado, Sheryl Pablo Abarquez, Ericson P Dilag, Criselda Bautista, Peter Craig, Katie Hampson, Nai Rui Chng

**Affiliations:** 1 MRC/CSO Social and Public Health Sciences Unit, University of Glasgow, Glasgow, UK; 2 School of Biodiversity, One Health & Veterinary Medicine, University of Glasgow, Glasgow, UK; 3 Field Epidemiology Training Programme Alumni Foundation Inc. (FETPAFI), Quezon City, Philippines; 4 Municipal Health Office, Alcantara, Philippines; 5 Department of Agriculture—Regional Animal Disease Diagnostic Laboratory (RADDL) MIMAROPA, Naujan, Philippines; 6 Research Institute for Tropical Medicine, Muntinlupa, Philippines

**Keywords:** Rabies, Global Health, Health systems, Philippines, Qualitative study

## Abstract

**Background:**

Zoonotic disease outbreaks are a growing global health concern, highlighting the need for tools to strengthen outbreak responses. Process mapping is a systems thinking approach that allows the complex multilevel, multisectoral network of actors and processes involved in an outbreak response to be clearly visualised and analysed using a One Health lens. We applied process mapping to a rabies outbreak in the previously rabies-free Philippine province of Romblon to demonstrate its utility.

**Methods:**

Key stakeholders were interviewed to inform the development of a provisional map of the actors, processes and challenges involved in the outbreak response. During a facilitated workshop, stakeholders worked in multisectoral, multilevel groups to refine the map and co-develop solutions to the challenges identified.

**Results:**

Process mapping revealed multiple challenges within rabies surveillance and outbreak response, particularly relating to case testing, recording and reporting, as well as intersectoral communication, community sensitisation and dog vaccination. Many of these issues were exacerbated by structural factors such as the Philippines’ archipelagic geography and decentralised governance of animal and human healthcare delivery. Broader issues linked with rabies’ endemic and neglected status were also identified: insufficiently One Health-orientated national guidance on outbreak declaration and response; resource constraints exacerbated by political devolution; and persistent weaknesses in intersectoral coordination and data sharing, compounded by gaps in international guidance on rabies and One Health practice. Suggested solutions that were possible to implement quickly with minimal resources, such as staff training, were undertaken. Recommendations requiring structural change, like enhancing reporting systems, were communicated to the provincial, regional and national government as policy briefs.

**Conclusion:**

Our research demonstrates the potential value of process mapping for enhancing One Health disease surveillance and outbreak response, strengthening health systems, and improving preparedness. To address the more systemic challenges revealed, outbreak response guidance must be improved, for rabies specifically and within One Health frameworks more generally, and the implications of political devolution on the control of neglected tropical diseases should be explored. These steps will be essential to achieving the global target of zero human deaths from dog-mediated rabies by 2030.

**WHAT IS ALREADY KNOWN ON THIS TOPIC**

**WHAT THIS STUDY ADDS**

- This study demonstrates the usefulness of process mapping in analysing and enhancing a zoonotic disease outbreak response and provides guidance on how to apply the approach in other contexts.
- Process mapping generated critical insights with the potential to enhance rabies surveillance and outbreak response in the Philippines and beyond. Our findings suggest that improvements in case diagnosis, recording and reporting—across both animal and human health sectors—must be paired with stronger intersectoral coordination, sustained dog vaccination and more effective community sensitisation.

**HOW THIS STUDY MIGHT AFFECT RESEARCH, PRACTICE OR POLICY**

- Our findings underscore the urgent need to develop national and international guidance for responding to rabies outbreaks, with attention given to the value of rapid diagnostic testing, the importance of proactive responses to animal cases to prevent human spillover, and the need for clear pathways for outbreak declaration and reporting.
- This study also highlights the potential impact of political devolution on the control of already neglected diseases, underscoring the need for further research on its implications for One Health implementation and governance.

## INTRODUCTION

 Infectious diseases are estimated to account for 43.7% of human deaths in low-income countries.^1^ Approximately 60% of known infectious diseases and up to 75% of emerging infectious diseases originate in animals.^2^,^4^ These diseases have profound consequences for human and animal health, as well as for economies.

As a result, it is vital that we learn how best to respond to such outbreaks that are increasingly part of the global health landscape. A concerning example of one such zoonotic disease is rabies. There has been a surge in rabies outbreaks across Southeast Asia over the last 20 years. Rabies emerged across multiple provinces in Indonesia that were historically rabies free^5^ and was detected for the first time in Timor-Leste in 2024.^6^ Rabies also recently re-emerged in Malaysia after over 60 years of rabies freedom.^7^ Efforts to contain these outbreaks have mostly failed.

An outbreak is broadly defined as a higher than anticipated number of cases of a disease in a particular location over a specific period.^8^ For zoonotic diseases such as rabies, a single human case in a disease-free area can constitute an outbreak.^9^ Moreover, early detection of zoonotic pathogens in animals creates a vitally important window for intervention.^10^ Swift containment of outbreaks in animals is one of the most effective strategies to reduce spillover risk and protect human health.^11 12^ However, operationalising a timely and effective outbreak response is challenging under any circumstances, but particularly difficult for zoonoses. This is because a truly effective response to a zoonotic outbreak requires a ‘One Health’ approach involving intersectoral communication and collaboration between animal and human health sectors^13^, as well as coordination across other departments and levels of government. This integrated approach is not practised in many settings.^14 15^

To support countries in translating One Health principles into practice, the Quadripartite has developed the One Health Joint Plan of Action (OH JPA),^16^ which outlines priority pathways for strengthening One Health capacities. However, translating these priorities into improved outbreak preparedness requires practical and participatory approaches that facilitate and enhance intersectoral working.^17^ Approaches commonly employed in the context of disease outbreaks include after-action reviews,^18 19^ simulation exercises,^19 20^ joint external evaluations,^21^ 7-1-7^22^ and process mapping.^23 24^ Process mapping is a systems mapping approach that has been used for decades to understand complex processes in business and engineering.^25^ More recently, it has been applied to health systems, where its alignment with core One Health principles, such as systems thinking, multisectoral collaboration and co-production of knowledge, makes it ideal for analysing and enhancing zoonotic outbreak responses.^23^

Unlike many participatory approaches, process mapping allows clear, step-by-step visualisation of the complex network of actors and processes within a system. This enables more granular analysis of outbreak response pathways and supports strengthening of key components such as timeliness and integrated surveillance and reporting.^23^ Stakeholders from different sectors can thereby develop a common understanding of the system and, using a One Health lens, identify bottlenecks and challenges (or ‘pain points’^23^) within the response, as well as potential solutions to those challenges. Process mapping is a time and resource-efficient approach that lends itself to use *during* an outbreak (as opposed to before or after). This allows stakeholders to respond rapidly in outbreak situations, capitalise on any increased support or funding and translate short-term momentum into long-term health system strengthening and improved future outbreak responses.^26^

Here, a rabies outbreak in the Philippines is used as a case study to explore the application of process mapping in analysing and refining an outbreak response. Rabies is a zoonotic disease, with more than 99% of human cases resulting from the bite of an infected domestic dog.^27^ Rabies has the highest case fatality rate of any known infectious disease (of nearly 100%)^28^ and still kills thousands of people each year in low-income and middle-income countries,^29^ with 200–300 deaths recorded annually in the Philippines.^30^ Romblon, our study province, was declared rabies-free in 2019, following a 7-year period with no detected cases.^31^ However, since September 2022, it has been in the grip of a rabies outbreak.^31^ This is reflective of the broader context with the Philippines seeing an upsurge of human cases since 2020^32^ and an escalation in the spread of rabies generally across Southeast Asia.^5^,^733^

Rabies is an excellent case study for process mapping because of the nature of both its surveillance and prevention. As a zoonotic disease, rabies requires extensive intersectoral communication and collaboration for effective surveillance. Its fatal yet preventable nature necessitates rapid administration of postexposure prophylaxis (PEP) to prevent death after exposure. Control of rabies involves addressing the source through vaccination of dogs. Together, these characteristics add complexity to rabies outbreak management, making it a useful example for demonstrating the utility of the process mapping approach.

Process mapping the response to a rabies outbreak will also benefit the global strategic plan (Zero-by-30) to eliminate human deaths from dog-mediated rabies by 2030.^34^ As this collaborative, country-centric initiative works towards rabies elimination, effective surveillance and enhanced outbreak responses will be required to maintain and expand rabies-free areas. By mapping the response to an outbreak in a previously rabies-free province, we will learn more about the challenges that elimination efforts will likely bring, both locally and globally.

Our objectives when undertaking process mapping were to: (1) explore how the outbreak detection and response unfolded; (2) identify which elements could be improved; (3) co-develop solutions to these issues to strengthen the response and the (One) health system more broadly; and (4) demonstrate the value of the process mapping approach in the context of a zoonotic disease outbreak. Using this approach, we identified several aspects of the outbreak response that require improvement. Many of these findings have broader implications for the control of rabies and other zoonotic diseases and for One Health governance more generally.

## Methods

### Study site

**Figure 1:**
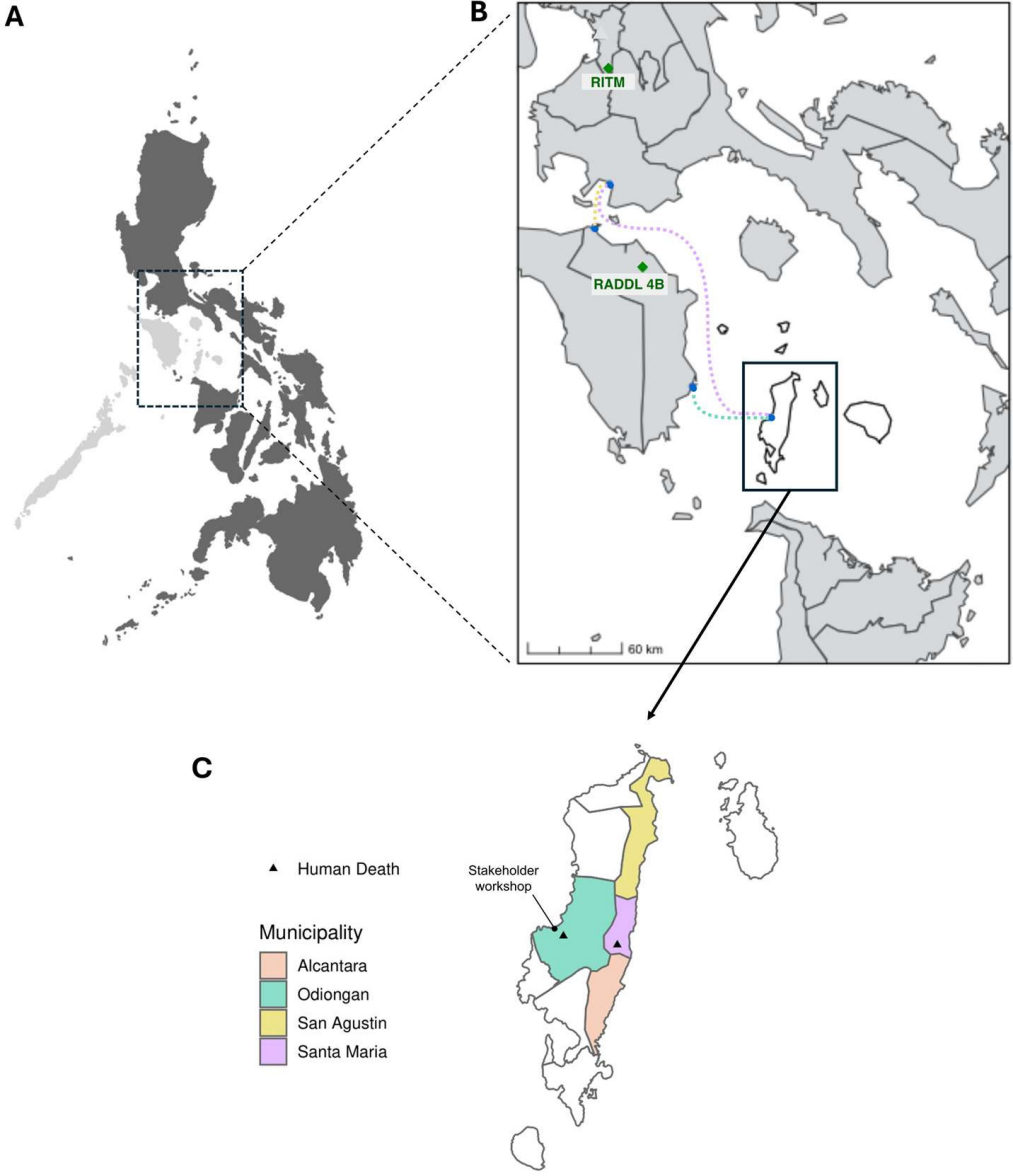
Location of outbreak and process mapping activities in the province of Romblon in the Philippines. (A) The Philippines showing the MIMAROPA region, also known as Region IV-B (light grey). (B) MIMAROPA region showing Romblon province (white), the Regional Animal Disease Diagnostic Laboratory (RADDL) in Oriental Mindoro and the Research Institute of Tropical Medicine (RITM) in Manila. Dashed lines show the main ferry routes used to transport human and animal rabies samples from Tablas Island to RADDL and RITM. Ferries leaving Tablas Island typically only depart once daily. (C) Tablas Island with key municipalities coloured and the locations of human deaths shown (black triangles). The outbreak was first detected in Santa Maria in November 2022, but the index case (30 September 2022) was ultimately determined to have occurred in Alcantara. Santa Maria and San Agustin municipalities both declared a state of emergency over the course of the outbreak.

This study focused on Tablas Island in the province of Romblon in the MIMAROPA region of the Philippines (figure 1). Tablas Island comprises nine municipalities and has a human population of around 175 000^35^ and an estimated dog population of between 17 000 and 58 000.^31^ There are seven Animal Bite Treatment Centres (ABTCs) on the island and one Municipal Agricultural Office (MAO) per municipality. ABTCs are government-run clinics in the Philippines that provide PEP (vaccination ± immunoglobulin), usually free of charge. MAOs oversee the local implementation of agricultural services and are responsible for prevention and control of animal diseases. Animal samples are sent either to the Regional Animal Disease Diagnostic Laboratory (RADDL) (part of the Department of Agriculture) in the neighbouring province of Oriental Mindoro, or to the national reference laboratory for rabies at the Research Institute for Tropical Medicine (RITM) in Manila for confirmatory testing.

Mass dog vaccination campaigns have been conducted annually in Romblon province since 2011 but estimated coverage rates have never surpassed 40% and efforts were suspended entirely in 2020 and 2021 due to COVID-19 pandemic restrictions.^31^

**Figure 2:**
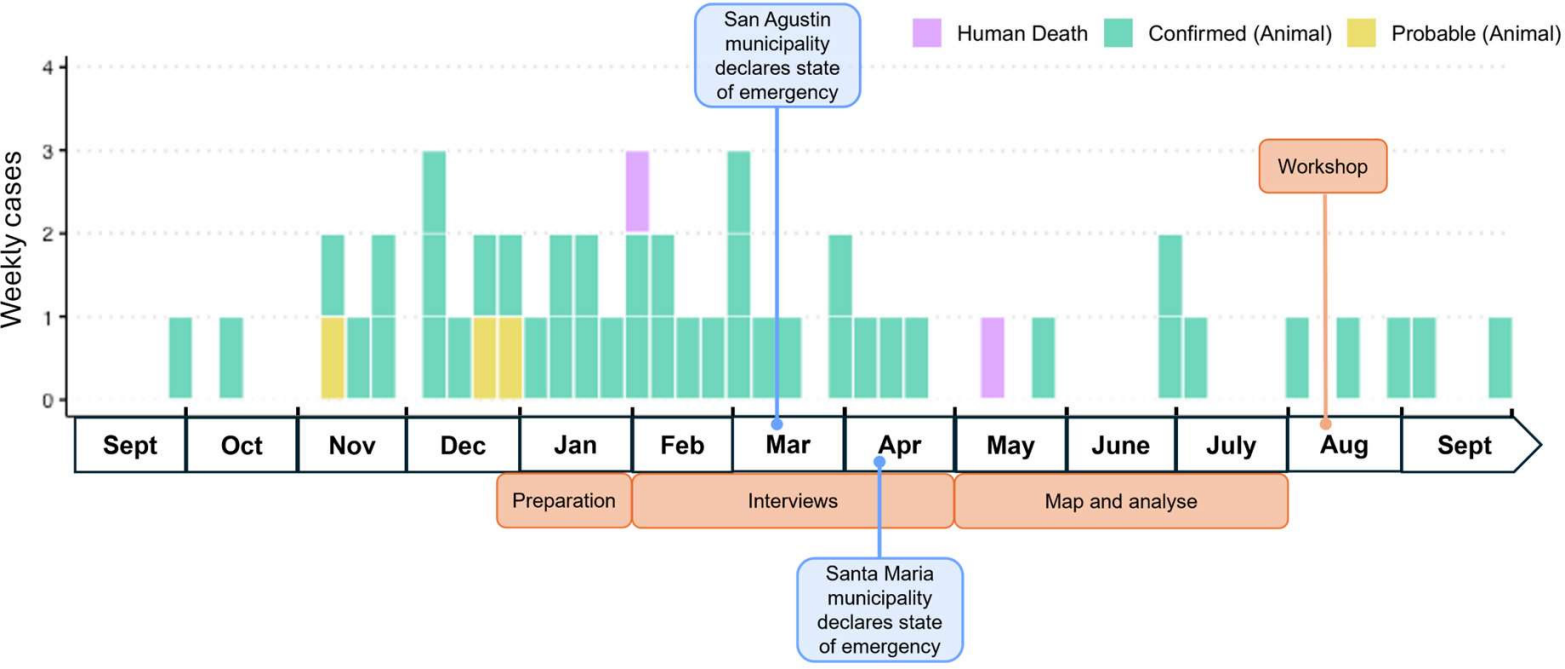
Timeline of outbreak and process mapping activities on Tablas Island, Romblon province between September 2022 and September 2023. Animal cases were confirmed by fluorescent antibody testing. Human cases were confirmed by polymerase chain reaction (PCR) testing. Animal cases are included as ‘probable’ cases if they had a history consistent with rabies, but testing was not performed. Human cases are dated by month of death; animal cases are dated by month of biting incident (if known), otherwise by sample collection date.

On 30 September 2022, an individual was bitten by a dog that was later confirmed to be positive for rabies (figure 2). A further 39 confirmed animal rabies cases and two human rabies deaths occurred in Romblon in the period leading up to the process mapping workshop (held on 10 August 2023). At the time of writing, the outbreak was still ongoing in the province, although cases have declined considerably.

The SPEEDIER project (Surveillance integrating Phylogenetics and Epidemiology for Elimination of Disease: Evaluation of Rabies Control in the Philippines) began in 2018 with a focus on piloting Integrated Bite Case Management (IBCM) in two provinces (Romblon and Oriental Mindoro). IBCM is a World Health Organisation (WHO)-recommended One Health approach to rabies surveillance that strengthens communication between human and animal health workers to improve rabies detection and reporting.^36 37^

### Study design

We employed a stakeholder-engaged process mapping approach as described by Durski *et al*,^23^ comprising four stages: preparation, interviews, map and analyse, and validate and co-create.

#### Preparation

The preparatory stage aims to identify key operational challenges present in the outbreak response through discussions with senior leadership, allowing the objectives of the process mapping exercise to be clearly defined. At this stage, it is helpful to identify metrics that allow challenges to be quantified (eg, sample turnaround times) and facilitate future evaluation of the success of the outbreak response redesign.

We conducted this stage through a mixture of in-person and virtual meetings with SPEEDIER research staff.

#### Interviews

This stage involves interviews with relevant stakeholders to learn about rabies surveillance and the outbreak response in detail and identify bottlenecks and challenges (or ‘pain points’^23^) in the system.

In-country collaborators assisted with the identification of appropriate stakeholders and care was taken to ensure a diverse participant group, reflective of the multisectoral, multilevel nature of the study topic. Interviewees included municipal agricultural staff, the Provincial Veterinary Officer (PVO), healthcare staff, laboratory staff and regional representatives from the Department of Health. A topic guide (online supplemental file 1) was developed and used during the semistructured interviews, consisting of mostly open-ended questions relating to stakeholders’ experiences responding to the outbreak and any challenges or successes they encountered. We conducted most interviews in person in Romblon in February 2023. For stakeholders not available during this period, or who did not reside in Romblon, interviews were conducted remotely.

#### Map and analyse

During stage 3, information gathered during stakeholder interviews is used to draft a provisional map showing the steps involved in the outbreak response and the pain points within the system.

We created our map digitally using BioRender, a web-based application used to create scientific figures and diagrams. We deviated somewhat from the standard notation often used for process mapping^38^ to make our map as intuitive and user-friendly for workshop participants as possible (a standard notation version of the map is provided in online supplemental file 2). Given the nature of rabies transmission, we focused our map on the steps that unfold after a potentially rabid dog bites a human. The map was annotated to show the pain points present within the system, highlighted explicitly or implicitly by stakeholders.

#### Validate and co-create

**Figure 3:**
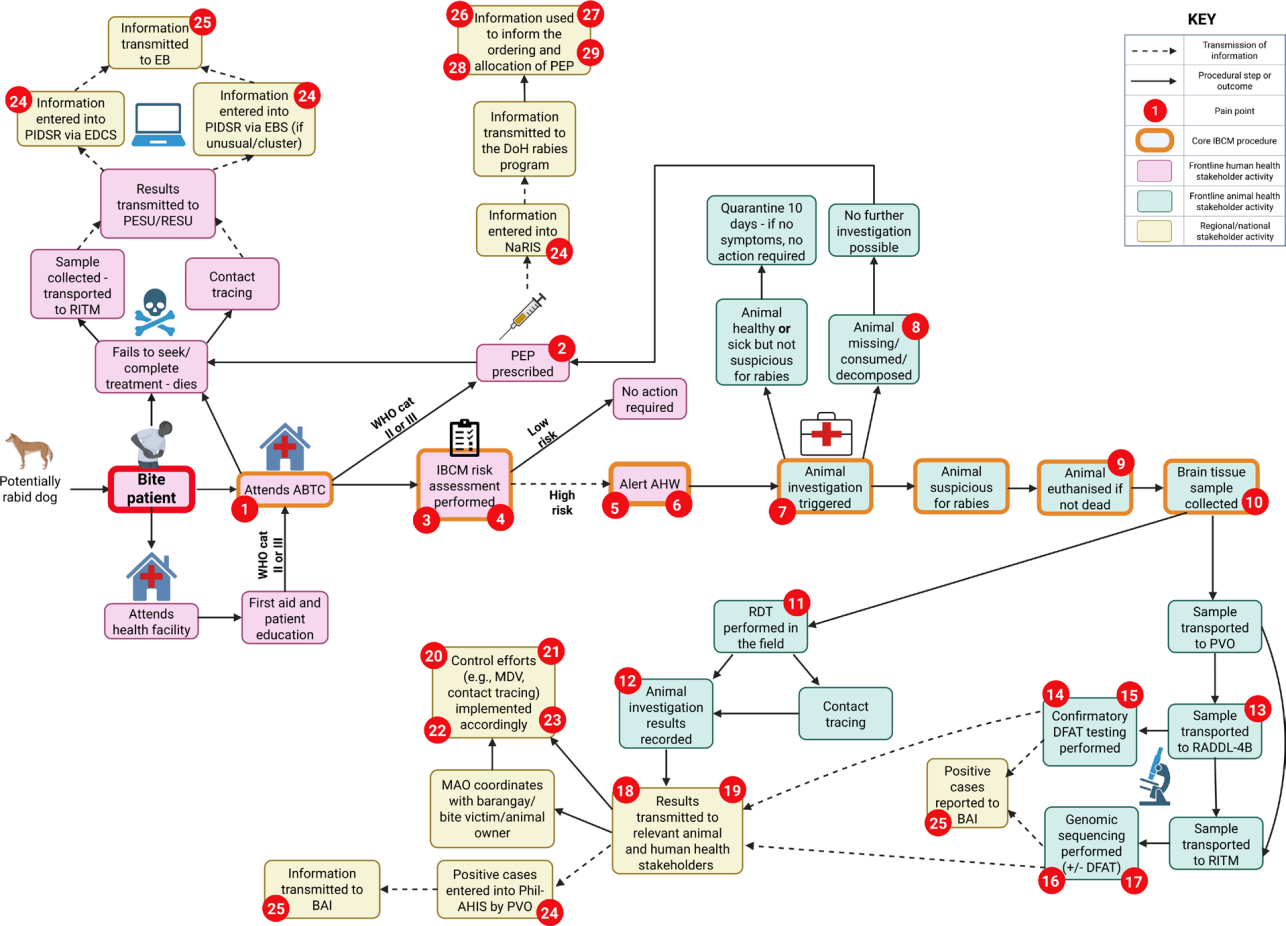
Refined process map developed from stakeholder input during the workshop on Tablas Island. To maximise clarity for participants, we adapted standard process mapping notation. Given the nature of rabies transmission, the map focuses on the steps that unfold after a potentially rabid dog bites a human (red ringed box). However, these processes can also be triggered by the identification and investigation of a potentially rabid animal, without the involvement of a bite patient. Orange ringed boxes denote core IBCM procedures. Numbered red circles denote pain points identified by stakeholders. Purple, green and yellow shading indicates how we divided the map for use by different stakeholder groups during the workshop. Icons (eg, dog, bite patient, health facility, etc.) were included to support map interpretation by workshop participants. Some minor edits have been made to the map by the author to improve clarity and accuracy. Example pain points are presented in table 1, and the full list is provided in online supplemental file 3. ABTC, Animal Bite Treatment Centre; AHW, Animal Health Worker; BAI, Bureau of Animal Industry (Department of Agriculture); DFAT, Direct Fluorescent Antibody Test; DOH, Department of Health; EB, Epidemiology Bureau (DOH); EBS, Event-Based Surveillance; EDCS, Epidemic-prone Disease Case Surveillance; IBCM, Integrated Bite Case Management; MAO, Municipal Agriculture Office; MDV, Mass Dog Vaccination; NaRIS, National Rabies Information System; PEP, post-exposure prophylaxis; PESU/RESU, Provincial/Regional Epidemiology and Surveillance Unit; Phil-AHIS, Philippine Animal Health Information System; PIDSR, Philippine Integrated Disease Surveillance and Response; PVO, Provincial Veterinary Office; RADDL, Regional Animal Disease Diagnostic Laboratory; RDT, Rapid Diagnostic Test; RITM, Research Institute for Tropical Medicine; WHO cat. II/III, WHO exposure categories II and III.

During the final workshop stage, stakeholders come together to review and refine the map (figure 3) and pain points (table 1) to ensure they reflect the reality of the response (full list of pain points provided in online supplemental file 3). They are asked to suggest actionable solutions to the pain points identified and allocate these to the relevant stakeholders for actioning, with appropriate time frames and metrics identified as needed (online supplemental file 4).

**Table 1:** Examples of pain points and actionable solutions suggested by stakeholders

Pain point		Actionable solutions
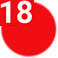	Failure of bite victim to present to ABTC for PEP.	Increase the production and sharing of rabies awareness social media materials. Incorporate educational talks on rabies into school curriculum and family development sessions. Re-evaluate the role of traditional healers in the patient’s PEP-seeking journey. Issue an advisory to all referring health facilities regarding ABTC opening hours and the referral process.
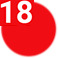	Animal investigations not carried out.	Provide ABTC staff with refresher training on One Health communication and the identification, recording and reporting (to AHWs) of ‘high risk’ bites. Create a protocol clarifying the responsible AHW in instances where a bite occurs in a municipality without an ABTC. Provide AHWs refresher training on One Health communication and the investigation of ‘high risk’ bites (including how to perform rapid testing in the field). Distribute an official waiver form (*Kasunduan sa Pagsuko ng Aso*) to all AHWs allowing the owner to relinquish responsibility of the biting animal to the AHW.
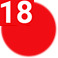	Turnaround time between sample collection and result dissemination too long to allow timely response.	Ensure that shifts are scheduled such that there is always at least one vet available at RADDL to process samples. Begin using the already established human sample transport chain (as used for COVID, polio, measles etc.) to transport animal rabies samples from the hospital to RITM.
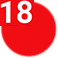	Investigation and laboratory results not communicated to healthcare staff and other parties.	Create a protocol and provide training clarifying how and to whom the results of animal sample testing should be shared, considering different possible scenarios (positive vs negative result, human bite victims vs no human bite victims). Create and enforce a protocol outlining how (and to which higher authorities) human and animal cases should be reported. Reactivate the intersectoral Provincial and Municipal Rabies Committees.

These examples were chosen to show the range of issues identified by stakeholders. A full list of pain points and the policy documents produced based on the suggested solutions are provided in online supplemental file 3.

ABTC, Animal Bite Treatment Centre; AHW, Animal Health Worker; PEP, post-exposure prophylaxis; RADDL, Regional Animal Disease Diagnostic Laboratory; RITM, Research Institute for Tropical Medicine.

We conducted our workshop in Odiongan in Romblon province (figure 1C). It was attended by stakeholders from all municipalities and included three municipal health officers, four municipal agriculturalists, the rabies coordinator from the PVO, a representative from the Provincial Health Office, and disease surveillance officers (DSOs) from the Provincial Department of Health. Regional stakeholders from the Centre for Health Development MIMAROPA, RADDL and RITM joined the workshop virtually. The workshop was facilitated by the research team, with some researchers present in person and others facilitating virtually.

Due to the extensive nature of our process map, we split it into three segments for analysis. Workshop participants initially examined each segment in multisectoral groups to enable a One Health approach to the analysis. They were then divided into three sector-based groups: frontline human health workers, frontline animal health workers, and regional level stakeholders (from both government and laboratory settings). Each group was allocated the segment of the map most relevant to their sector. Within these groups, participants agreed on a final list of pain points and proposed actionable solutions.

Data collected during the stakeholder workshop included audio and video conference recordings of group sessions, notes taken by facilitators (online supplemental file 4) and the map and pain points edited by stakeholders. The data collected during the workshop were used to refine the provisional map and pain points. These data were also used to produce a series of documents for stakeholder use, including an executive summary and urgent appeal (sent to high level provincial stakeholders) (online supplemental file 5), a more detailed summary of findings and recommendations (sent to all workshop participants and those identified as ‘responsible agencies’) (online supplemental file 6) and individually tailored letters to each responsible agency highlighting specific actionable solutions identified as within their remit.

Furthermore, to inform the conclusions of this study, data analysis was conducted by the first author using a thematic analysis approach.^39^ All interview and workshop transcripts, plus government documents, were read for familiarisation and any initial concepts that emerged were noted. The data were then coded, ensuring that each data item relevant to our research objectives received a code. Using an inductive approach, codes were collated into broader themes, which were then grouped into categories. These categories provided the foundation for the study’s conclusions, which were subsequently reviewed by the authors (who represent a diverse range of professional backgrounds and include local stakeholders) and workshop facilitators to ensure accuracy. In addition, government and project (SPEEDIER) operational data were analysed to help support analysis. Drawing on multiple data sources enabled triangulation, allowing us to verify that findings were consistent across sources and thereby strengthening the validity of our conclusions.

## Results

### Outbreak response description

The outbreak response process map developed with stakeholders is presented in figure 3 and described further in this section. IBCM played a crucial role in the Romblon outbreak response and much of our process mapping centred around learning about its operationalisation in this context (see ‘core IBCM procedures’ in figure 3).

The outbreak was first detected on 21 November 2022, when the use of IBCM and field testing led to the identification of a rabid dog involved in a biting incident 3 days earlier. This prompted the testing of previously collected (frozen) dog brain tissue samples, revealing the index case to have died 7 weeks earlier, on 30 September 2022.

During the study period, nurses at primary healthcare facilities provided first aid, assessed patients based on WHO wound severity guidelines^40^, and referred them to ABTCs for PEP. At ABTCs, staff administered PEP, recorded bite cases and PEP data in the National Rabies Information System (NaRIS) and assessed whether animal health counterparts should be notified (depending on the likelihood of rabies in the biting animal). High-risk bite cases were reported to animal health workers (AHWs) or DSOs by phone or Facebook Messenger. AHWs then attempted to locate the biting animal for euthanasia (if suspicious for rabies) or quarantine (if seemingly healthy). The heads of suspect cases were sent to the PVO before being sent on to RADDL or RITM for confirmatory testing. In some cases, AHWs performed field testing using a rapid diagnostic test (RDT). Confirmed cases were reported to the Bureau of Animal Industry by the PVO and/or staff at RADDL via the Philippine Animal Health Information System (Phil-AHIS) and reported to relevant animal and human health stakeholders.

Human rabies cases were diagnosed by polymerase chain reaction (PCR) testing at RITM and were entered into the Philippine Integrated Disease Surveillance and Response (PIDSR) system by Epidemiology and Surveillance Units (within 24 hours given the notifiable nature of rabies) via the Epidemic-prone Disease Case Surveillance (EDCS) system and transmitted to the Epidemiology Bureau. Due to Romblon’s previously rabies-free status, human cases were also entered into PIDSR via the Event-based Surveillance (EBS) system, which is used to capture unusual disease events or clusters of cases.

Some additional measures beyond routine IBCM were taken in response to the outbreak. The first confirmed animal case prompted ring vaccination of 66 dogs in the affected area and a state of emergency declaration by the affected municipality (Santa Maria). Contact tracing and vaccination of exposed humans and animals by local animal and human health workers were also conducted after the first confirmed case, and to varying degrees after subsequent confirmed animal cases. The first confirmed human case occurred 2.5 months later in the same municipality and resulted in an education and awareness campaign in the affected *barangay* (village) and latterly, a declaration by the governor that, in accordance with the Anti-Rabies Act of 2007 (Republic Act No. 9482), the mayors of all municipalities should allocate funds for dog vaccination. However, despite this, vaccination levels across the province remained uneven and generally low. Additional intersectoral efforts included convening the Romblon Provincial Rabies Committee, declaring a state of emergency in San Agustin municipality, and holding coordination meetings between municipal agriculturists, mayors, municipal councils and the provincial veterinarian.

### Outbreak response challenges

The creation and analysis of our process map (figure 3) identified 29 pain points across eight categories:

**Staffing shortages** hindered patient risk assessments, animal investigations and sample transport. ABTC staff lacked time to record and communicate case details, and overburdened AHWs were sometimes unable to complete investigations. Furthermore, the rabies coordinator at the PVO was often engaged in other duties and unable to prioritise the sending of rabies samples.

**Ineffective training** led to errors in IBCM risk assessment and data entry by ABTC staff. Despite efforts to emphasise the importance of the bite circumstances and signs of animal rabies**,** it was reported that staff often based risk evaluations on outdated WHO guidelines that focus only on wound severity and not risk associated with the biting animal. Many AHWs lacked confidence in brain tissue sampling, reducing field testing and leading to incomplete animal investigations.

**Resource constraints** resulted in insufficient access to personal protective equipment, leaving many AHWs unable to safely perform brain tissue sampling and rapid testing, further reducing rates of field testing. In some cases, lack of access to RDTs compounded this issue. Due to MAO budget and cold storage constraints, AHWs were unable to send samples directly to the laboratory. Routing samples through the PVO created additional workflow issues (see below) and resource constraints at the PVO often necessitated samples being dispatched to the laboratory in weekly batches, contributing to a mean turnaround time of 12 days. A broken fluorescent microscope at RADDL for much of the study period (October 2022 to May 2023) required samples to be transported longer distances to RITM for confirmation by direct fluorescent antibody testing (DFAT). Dog vaccination was also constrained by funding following the Mandanas-Garcia ruling, which devolved responsibility for local animal and human healthcare service delivery, including vaccine procurement, to local government units (LGUs).^41^ This policy change in April 2019 abruptly ended the supply of animal vaccines to the PVO, which, combined with inadequate local budgets to purchase vaccines independently, resulted in very limited dog vaccination activities and insufficient coverage. Periodic stockouts at ABTCs meant some patients had to purchase vaccines from private pharmacies, incurring out-of-pocket costs of approximately ₱1600–1950 (US$27–34) per vial (with a new vial required for each dose unless vial sharing occurs), creating accessibility challenges.

**Workflow inefficiencies** impacted sample transport, testing and results reporting. Sending samples via the PVO extended turnaround time, especially when the rabies coordinator was unavailable (see *staffing shortages*). These delays were compounded by long interisland journeys and infrequent ferries (figure 1B). Although RADDL performed RDTs on the samples they received prior to forwarding them on to RITM, staff believed they were not legally allowed to release RDT results until confirmed by RITM, forfeiting the opportunity for earlier action. The typical turnaround time for DFAT at RITM was 3 working days, counted from the receipt of the specimen in the rabies laboratory until the result was ready for release. Sample receiving was available 24/7, but testing was conducted only on working days. Result release was reported to occasionally be delayed by missing or incomplete case investigation forms, and there were reported instances of result forms being issued with incorrect information due to staff data-extraction errors. Finally, different sample naming conventions used by AHWs, RADDL, RITM and SPEEDIER meant a single sample would typically end up with four identifiers, complicating analysis.

**Patient/community-related issues** posed further challenges. Some patients failed to present for (or complete) their course of PEP due to a lack of awareness, financial constraints, preference for traditional medicine or logistical difficulties. High patient volumes—over 30 bite patients per day at the busiest ABTCs, including new and returning bite patients—prevented staff from conducting follow-up to ensure completion of PEP courses. Community-related challenges made some animal investigations challenging. AHWs encountered difficulties safely euthanising biting animals due to crowd control issues and/or local police refusing to assist by shooting the animal, citing previous public backlash. In other cases, community members killed and buried the biting animal prior to investigation, compromising sample quality. During the study, two human deaths from rabies were confirmed. Neither patient sought PEP after being bitten, demonstrating insufficient reach and/or effectiveness of community sensitisation (education) efforts.

**Intersectoral communication issues** resulted in unreported high-risk bites and incomplete dissemination of animal investigation results. Reports of human rabies deaths were often not shared with relevant agencies, particularly within the animal sector.

**Ineffective and siloed surveillance systems** further impacted the response. Multitudinous—and in some cases poorly functioning—health information systems overwhelmed already overburdened frontline workers, resulting in frequent failures to log epidemiological data (such as rabies exposures and PEP administration into NaRIS), impacting the completeness of regional and national records. Participants were familiar with the EDCS and EBS systems but referred to them only in the context of reporting human rabies deaths (not animal cases), consistent with existing government guidance. Although both human deaths (and many confirmed animal cases) were reported via official channels, no formal outbreak declaration was made and subsequent support from regional and national stakeholders was insufficient to enable the province to contain the outbreak.

**Lack of leadership and guidance** exacerbated the impact and extent of the outbreak. The lack of formal outbreak declaration likely impacted public awareness and urgency in seeking PEP in the event of a potential exposure. An official outbreak declaration would have facilitated the work of AHWs, by making the public (and other groups such as police) more receptive to necessary control measures, such as dog vaccination and the confining, euthanising and/or testing of suspect animals. An outbreak declaration would have also likely helped facilitate coordination (and potentially funding) of comprehensive dog vaccination across the province.

A related finding was the lack of national guidance on the declaration and management of rabies outbreaks. While some participants reported that the PIDSR Manual of Procedures and the Mandatory Reporting of Notifiable Diseases and Health Events of Public Health Concern Act (Republic Act No. 11332) provide declaration guidance, both resources offer only a broad directive—outbreak declarations must be …*supported by sufficient scientific evidence based on disease surveillance data, epidemiologic investigation, environmental investigation, and laboratory investigation*^42 43^ without further detail provided. The only specific criteria for declaring a rabies outbreak appear in the Anti-Rabies Act of 2007 (Republic Act No. 9482), which states: *When a confirmed [human] rabies case is reported in a declared rabies-free area, the LGU should declare an outbreak and conduct an immediate comprehensive response to control the spread of the disease*.^9^ This guidance was included in the 2012 National Rabies Prevention and Control Program (NRPCP) Manual of Procedures^44^ but omitted from the 2019 update. Notably, we found no official guidance for declaring a rabies outbreak in animals. Furthermore, although rabies outbreak response measures are mentioned in certain government documents,^44^,^46^ guidance is scattered, prioritises human cases, and, in the most recent version of the NRPCP Manual of Procedures,^45^ provides insufficient operational detail, especially with regards to the responsibilities of local level stakeholders. In 2021, the Global Alliance for Rabies Control published a ‘Rabies Rapid Response Toolkit’ for use in the Philippines.^47^ This document contains guidance on enacting a One Health response to various rabies triggers but does not give guidance on outbreak declarations and was not mentioned by any of our participants.

### Outbreak response solutions

In their sector-based groups, participants proposed actionable solutions to the final list of pain points (online supplemental file 3) and identified metrics and responsible agencies for many of these. For some solutions, it was possible to secure on-the-spot commitment to immediate implementation from participants. Others required higher level authorisation and further discussion regarding human and financial resource requirements.

The solutions (table 1) suggested by workshop participants fell into three main categories.

Local-level changes comprised improvements considered relatively straightforward to implement at municipal or provincial level. These included staff training; development of new forms and protocols; issuance of public advisories; intensified education and awareness campaigns; increased budget allocation for dog vaccination; and minor adjustments to animal sample transport and processing.

Higher level changes centred on more structural solutions that required implementation by provincial, regional and/or national stakeholders. Examples included training traditional healers to refer bite patients for PEP; using the human sample transport chain for animal rabies samples; refining case reporting systems; and clarifying outbreak declaration procedures.

The final category consisted of solutions to One Health collaboration and communication challenges. These included reactivating intersectoral rabies committees, integrating currently siloed surveillance systems, and strengthening aspects of IBCM operationalisation. Many IBCM-specific challenges and solutions related to training and were addressed through a SPEEDIER IBCM training session conducted in Romblon shortly after the workshop. There was strong consensus from stakeholders that the workshop itself was hugely valuable in enhancing One Health working, as it provided a unique opportunity to connect with colleagues across sectors and analyse challenges using a One Health approach.

## Discussion

### Main findings

Our findings indicate that, despite the presence of a One Health rabies surveillance system in this context, many elements of rabies surveillance and outbreak response required strengthening across sectors and levels. Operational issues included limited field testing and protracted delays prior to sending samples for laboratory diagnosis, resulting in delayed identification of rabid animals and impeding proactive public health decisions. Inconsistent case recording and limited intersectoral communication hindered monitoring, implementation of response measures and outbreak control. Low dog vaccination coverage prolonged this outbreak, and steps taken to sensitise communities regarding rabies risks were inadequate. Failure to declare an outbreak arguably led to preventable deaths in bite victims who might have otherwise sought care. Operational gaps were compounded by structural constraints, including insufficient national and international guidance on outbreak declaration and response, and the Philippines’ archipelagic geography and decentralised delivery of animal and human healthcare.

Proposed solutions included local-level improvements such as staff training, public advisories and streamlined sample processing; structural reforms such as enhanced reporting systems and clearer outbreak response guidance; and stronger One Health collaboration through improved data sharing, reactivated rabies committees and enhanced IBCM implementation.

Since rabies is widely regarded as an excellent ‘test case’ for analysing and strengthening One Health implementation,^48^ our process mapping findings highlight various challenges and opportunities countries like the Philippines will face when implementing a OH JPA at the national level.^16^

### Outbreak response

Our findings reveal gaps in the outbreak response, with a key shortcoming being the failure of the local government to officially declare an outbreak. Although limited engagement with senior leadership prevented us from determining definitively why no formal outbreak declaration was issued, insufficiently One Health-oriented government guidance—on what constitutes an outbreak and the pathway to declaration—likely contributed. This specific issue is not widely explored in other contexts, but policy frameworks for zoonotic disease control are often not well aligned with One Health principles, constraining effective outbreak detection and response in both high-income and low-income settings.^49^,^52^ Other reported barriers to outbreak declarations include concerns about trade and tourism impacts^53 54^ and technical and resource-related barriers.^55^ Fragmented or poorly integrated surveillance systems can also hinder outbreak declarations, especially for zoonoses.^55^ This gap points to a shortfall in the OH JPA Pathway 1 (governance, policy, legislation, financing and advocacy).^16^ While the Philippines has a national, multisectoral rabies coordination mechanism—the NRPCP—in place, its guidance on outbreak declarations and response was not sufficiently detailed or One Health-oriented to translate to clear, actionable direction for stakeholders working on the ground.^45^

The implications of not declaring an outbreak can be profound. Despite generally high levels of postbite healthcare seeking in the Philippines,^56^ reaching high-risk bite victims—who disproportionately come from marginalised communities with lower rates of health-seeking behaviour—and ensuring they receive life-saving PEP remains challenging.^30 32^ In this outbreak, both human cases sought traditional *tandok* treatment instead of PEP, reflecting a breakdown in OH JPA Pathway 2 (organisational and institutional development, implementation and sectoral integration) in risk communication and community engagement.^16^ Reliance on traditional healing persists among a concerning proportion of bite patients in the Philippines^57^, as well as in other endemic settings such as Nigeria,^58^ Nepal^59^ and Ethiopia.^60^ Publicly declaring outbreaks—whether in animals, humans, or both—helps heighten risk awareness among communities and professionals, encourages appropriate care-seeking and frontline response behaviours, and mobilises the resources required to mount an effective response, all of which are critical to limiting outbreak spread and impact.^15 61^

Despite confirmed animal and human deaths reported via surveillance systems such as PIDSR and Phil-AHIS, persistent resource constraints hampered the response. Regional and national stakeholders provided valuable technical support, for example by supporting laboratory confirmation of both human cases, which is notable given that human cases in the Philippines are mostly diagnosed clinically.^62^ However, following the Mandanas-Garcia ruling, these stakeholders could not substantially expand access to resources such as PEP and dog vaccines, and LGUs likewise struggled to allocate the additional funds required. More broadly, as in other contexts,^63^ animal health resource mobilisation in the Philippines appears strongly driven by economic and food-security considerations, as illustrated by African Swine Fever’s nationwide emergency designation^64^ and multibillion-peso swine recovery funding.^23^ It is also shaped by Global North agendas that prioritise mitigating health security threats to high-income countries, as reflected in international pressure to control highly^65^ pathogenic avian influenza^66 67^ even as rabies control remains chronically underfunded.^68^

As patient perspectives were not included in this study, we are unable to determine whether PEP shortages and out-of-pocket patient costs impacted health-seeking and clinical outcomes during this outbreak. Evidence from other parts of the Philippines suggests that while direct and indirect costs can act as barriers, they are far from the only determinants of care-seeking.^57^ Other factors, such as lack of awareness and reliance on traditional medicine—both highlighted in this study—also play out.^57 69^ What is clear, however, is the critical role of dog vaccination and the repercussions of underinvestment: the persistence of the outbreak underscores the consequences of inadequate vaccination coverage. Addressing the implications of political devolution, particularly the underprioritisation of animal-focused disease control measures, will be crucial if the Philippines is to overcome the financial barriers currently impeding progress along OH JPA Pathway 1.^16^

A further key finding was inconsistent intersectoral communication and coordination, which frequently undermines zoonotic outbreak responses.^70 71^ While the implementation of IBCM improved frontline intersectoral communication in this setting, limited coordinated action and fragmented data reporting remained persistent challenges within OH JPA pathways 2 and 3 (data and evidence, information systems and knowledge exchange).^16^ The NaRIS system illustrates these weaknesses: its poor functionality means it is now largely unused by frontline workers to report rabies exposures and PEP use. Implementing OH JPA-aligned strengthening measures, alongside use of Tripartite tools—such as the Multisectoral Coordination Mechanism Operational Tool^72^ and the Surveillance and Information Sharing Operational Tool^73^—by a national steering committee, could help identify these gaps and support the development of an actionable plan to address them. These tools employ, among others, systems mapping approaches to achieve their objectives and would build on our findings. Rabies’ dual nature as both endemic (in many settings) and outbreak-prone makes it well suited to this approach: investments in ‘peacetime’ coordination and information sharing can strengthen ‘wartime’ outbreak responses and, over the longer term, improve health system resilience by enabling more agile collaboration during future crises.^26^

### Broader implications for rabies

The challenges identified reflect wider weaknesses in rabies surveillance and guidance globally, with implications for detection, reporting and response beyond the Philippines, as well as for achieving the ‘Zero by 30’ goal.^34^ Several OH JPA pathway 2 implementation challenges were evident.^16^ Maintaining frontline worker proficiency in IBCM risk assessment proved challenging. Risk assessments were performed incorrectly despite regular training and updates to WHO guidance,^74^ highlighting the difficulty of translating internationally developed guidelines into routine practice, and replacing entrenched, outdated practices. Sustaining confidence in field testing also proved difficult. Progress towards rabies elimination will inevitably involve reduced testing, as case numbers drop. Our findings suggest that this decline in testing could undermine practitioner confidence. To maintain and expand rabies-free areas, finding ways to sustain frontline worker proficiency in performing these surveillance tasks will be crucial to ensure incursions and outbreaks are detected and dealt with expediently.

Stakeholders had concerns regarding the legality of sharing and acting on RDT results, due to a lack of national protocols on their use. This reflects another OH JPA pathway 1 governance challenge^16^ but was compounded by the absence (at the time of data collection) of guidance from international organisations. Recently published guidance by the World Organisation for Animal Health (WOAH) represents progress in this area^75^ but further work is required to improve, validate and standardise these tests or gaps will continue to constrain surveillance in settings with limited laboratory access.

Due to its endemic nature in many settings, rabies is often not recognised as outbreak-prone, despite evidence to the contrary.^6 7 33 76^ This perception, combined with weak surveillance, contributes to under-reporting. For example, although the Philippines is a member of WOAH and rabies is a notifiable disease, no rabies outbreaks from the Philippines appear in WOAH’s World Animal Health Information System events management database since its launch in 2005 (as of September 2025).^77^ On the human side, cases recorded in the PIDSR system could be reported to WHO via the International Health Regulations (IHR) framework,^78^ but based on local interpretation of the IHR Annex 2 decision instrument, this did not happen for this outbreak. Rabies is not among the diseases requiring mandatory WHO notification, and generally speaking, global reporting is widely acknowledged to be low.^79^ The perception of rabies as non-outbreak prone has likely contributed to the relative lack of international outbreak response guidance compared with other pathogens,^80 81^ with downstream effects on the quality and availability of local and national guidance (including in the Philippines) and implications for rabies surveillance and control. Given its geographic spread across Southeast Asia and the increasing risk of incursions into countries such as Australia,^82^ strengthening of rabies outbreak detection, reporting and control is warranted.

### Broader implications for One Health implementation and governance

Existing One Health guidelines emphasise multisectoral collaboration and surveillance strengthening and, in one case, specify triggers for activating contingency plans.^15^ However, they typically provide limited explicit operational guidance on the country-level decision pathways and escalation procedures through which endemic zoonoses can be formally recognised and declared as outbreaks. As our process mapping illustrates, these ‘grey areas’ can translate into uncertainty and delays at the point of local decision-making.

Intersectoral governance mechanisms are central to effective and equitable One Health responses to health security emergencies such as zoonotic disease threats.^16^ While the Philippines has made strides at the national level (including the establishment of the Philippine Inter-Agency Committee on Zoonoses in 2011),^83^ the political economy of resource distribution and decision-making in the Philippines is increasingly shaped by political devolution, with implications for One Health policy and practice. Research has raised concerns regarding the impacts of the Mandanas-Garcia ruling on local health programmes. Although the ruling aims to increase the budgets provided to LGUs, poorer or smaller LGUs may gain less usable funding and therefore struggle to allocate adequate budget for certain resource-intensive health services amid competing local priorities.^84^ From a One Health perspective, devolution may be particularly consequential because effective prevention of zoonoses such as rabies depends on sustained control in the animal reservoir.^34^ Devolution has fragmented accountability for mass dog vaccination across LGUs, making long-term investment in dog-focused prevention harder to coordinate at scale—even though these upstream investments yield downstream benefits through reduced human exposures and deaths.^85^

Further investigation is required to determine the specific effects on neglected disease programmes. Nevertheless, our findings suggest that a devolved health political economy, even where national-level One Health mechanisms are emerging, risks entrenching the neglect of certain diseases and may make coordinated, wide-scale control of these diseases more challenging. This is supported by evidence from other settings, such as Kenya and Indonesia, where local health service delivery, especially preventative care, has been negatively impacted by devolution.^86^ Given that rabies is already underfunded and deprioritised in favour of diseases considered to have greater economic impacts,^31^ maintaining rabies as a sustained cross-government priority—particularly through reliable financing for MDV—is essential in the wake of this ruling. Within internationally developed One Health guidance, greater attention should be given to how political devolution can reshape accountability, financing and operational performance for endemic, neglected zoonoses.

### Process mapping

Through this exercise, we learnt about the benefits, challenges and practical nuances of process mapping, informing its future application. The approach helped to elicit tacit knowledge not usually shared in a formal and systematic way and pinpointed where—and why—critical knowledge–practice gaps persist. Process mapping allowed participants to develop a collective and causal understanding of the intersectoral outbreak response and identify both operational and structural areas in need of improvement. Several operational issues could then be addressed rapidly through training and other actionable changes, strengthening the response and supporting longer-term system improvement.

The collaborative, workshop-based nature of process mapping fostered stronger relationships and communication between stakeholders from different sectors, aligning with evidence on the importance of intersectoral communication during outbreaks.^20 87^ Participants repeatedly noted how valuable (and unusual) it was to convene as a multisectoral, multilevel, whole-province group. Stakeholders found the experience so positive that the Centre for Health Development MIMAROPA subsequently requested support for a process mapping workshop to address rabies resurgence across the wider region. Conducted in February 2024, this workshop resulted in a region-wide multisector-supported dog vaccination campaign, among other positive outcomes. Furthermore, two municipalities in Romblon have since passed One Health ordinances (local laws) to improve the intersectoral response to future zoonotic outbreaks.

Our experience with process mapping differed from that of Durski *et al*^23^ in some ways. While Durski *et al*^23^ used process mapping to enhance responses to emerging diseases of international interest with support from the WHO, we applied the approach to an endemic neglected zoonosis, without the backing of an international organisation. Rabies’ neglected status made it difficult to secure buy-in from senior stakeholders and constrained efforts to secure additional resources. Although the process still proved very useful, this finding should be considered when using process mapping in similar contexts. We also invested substantial time in communicating findings to provincial, regional and national stakeholders through policy briefs, an additional follow-up stage not explicitly described by Durski *et al.*^23^

Despite numerous interviews with expert stakeholders, it was challenging to establish a clear, fully comprehensive picture of the outbreak response, likely reflecting the complexity of the government and health system structures and the need to incorporate both animal and human health systems. Process mapping nevertheless proved effective in creating a shared systems-level understanding and highlighted the critical need for streamlining.

This study adds to a growing body of evidence demonstrating the value of process mapping for analysing and strengthening responses to zoonotic outbreaks. While its utility has been shown in contexts such as Ebola, yellow fever, monkeypox^23^ and dengue,^88^ the approach remains underused and holds potential for wider application across diseases and settings. Process mapping targets operational bottlenecks and challenges in a highly efficient, focused manner (with the whole process often completed within the space of a week),^23^ compared with approaches such as OH-SMART, which are typically carried out over longer periods.^24^ This time-efficient approach makes it particularly well-suited to use during an outbreak. When paired with the 7-1-7 framework, process mapping can also support assessment and improvement of a country’s timeliness in detecting and responding to emerging events.^22^ By providing a detailed account of how process mapping was operationalised in this case study, we aim to support and facilitate its future use in diverse geographic and epidemiological contexts.

### Limitations

All interviews for this study were conducted by a single researcher, with translation support from other team members. With more researchers, additional interviews—particularly on human rabies cases and surveillance—could have enhanced the depth of the map. Support from an international organisation might have improved engagement with high-level stakeholders. Another key limitation was the absence of community members as participants. We had concerns that an overly large participant group would make it harder for individuals to engage productively. As our priority was to map institutional workflows, we elected not to include traditional healers and households of bite victims in this study. This limited the extent to which community perspectives are represented in our findings and their inclusion would enhance future research on this topic.

The study prioritised identifying system challenges to refine the response efficiently, although exploring positive aspects of the outbreak response would also have been valuable. The SPEEDIER project’s prior relationships with interviewees, and history of providing support for rabies activities, may have introduced social desirability bias. Additionally, logistical challenges posed by the country’s geography and climate, including typhoon-related delays, postponed the workshop by 8 weeks. While this prevented the rapid-response approach described by Durski *et al*, it allowed for extended data collection and outbreak monitoring. As our conclusions are based on a single outbreak response, findings may not generalise to other outbreaks. Finally, while the exercise led to several positive changes, resource challenges prevented full implementation of all suggested actionable solutions, and no formal evaluation of its outcomes was conducted.

## Conclusion

This study demonstrates the value of process mapping as a tool for analysing and improving responses to zoonotic outbreaks. By providing a clear visualisation of key actors and processes, it enabled the identification of weaknesses in a rabies outbreak response and supported the co-development of actionable solutions through One Health collaboration. Our study revealed issues associated with rabies’ endemic and neglected status and, in doing so, highlights some challenges and opportunities countries may face when implementing an OH JPA.^16^ Issues included insufficiently One Health-orientated national guidance on outbreak declaration and response, inadequate resource allocation exacerbated by political devolution, and persistent gaps in intersectoral coordination and data sharing. These constraints are compounded by blind spots in international guidance for rabies specifically and for One Health practice more generally.

Addressing these issues will require a two-pronged approach: (1) sustained engagement with high-level Philippine stakeholders to strengthen multisectoral collaboration, evidence-based policymaking and resource allocation within the country; and (2) the development and adoption of international rabies outbreak response guidelines, integrated into local policies, that support field testing and emphasise proactive responses to animal cases to prevent human spillover. More broadly, One Health frameworks should provide clearer operational guidance on the pathways through which endemic zoonoses are recognised, escalated and formally declared as outbreaks and should more explicitly consider how political devolution can reshape accountability and financing for neglected zoonoses, particularly those requiring sustained investment in animal-reservoir control. Taking these steps will support the Philippines and other countries in reaching the Zero by 30 goal^34^ and strengthen coordinated control of zoonotic threats more broadly. Moving forward, process mapping should be considered in other settings to support rabies outbreak management and strengthen responses to other zoonoses.

## Supplementary material

10.1136/bmjgh-2025-020482 online supplemental file 1

10.1136/bmjgh-2025-020482 online supplemental file 2

10.1136/bmjgh-2025-020482 online supplemental file 3

10.1136/bmjgh-2025-020482 online supplemental file 4

10.1136/bmjgh-2025-020482 online supplemental file 5

10.1136/bmjgh-2025-020482 online supplemental file 6

10.1136/bmjgh-2025-020482 online supplemental file 7

## Data Availability

All data relevant to the study are included in the article or uploaded as supplementary information.
