# Supplementary material for: Process mapping the One Health response to a rabies outbreak in the Philippines
Source: BMJ Glob Health. 2026 Apr 2;11(4):e020482. doi: 10.1136/bmjgh-2025-020482 (PMC13052803; doi:10.1136/bmjgh-2025-020482)
Supplement: online supplemental file 1 [file bmjgh-11-4-s001.pdf]

Supplemental file 1: Interview topic guide

The topic guide used during interviews conducted with stakeholders in Romblon in February 2023.

Probes

1. Can you please tell me your job title and give a short description of your role and responsibilities?
2. Can you tell me about your experiences dealing with the recent incursion in Romblon?
3. In what ways do you feel the incursion was dealt with well?
4. In what ways do you feel the incursion was not dealt with well?
5. What kind of hurdles/challenges have you encountered, if any, when trying to deal with the Romblon incursion? (SPEEDIER limitations or external factors?)
6. What role has SPEEDIER played in helping to detect the outbreak and in informing decision making regarding how best to respond to it?
7. What can we learn from this incursion/how can future incursions be dealt with better
