# Supplementary material for: Process mapping the One Health response to a rabies outbreak in the Philippines
Source: BMJ Glob Health. 2026 Apr 2;11(4):e020482. doi: 10.1136/bmjgh-2025-020482 (PMC13052803; doi:10.1136/bmjgh-2025-020482)
Supplement: online supplemental file 2 [file bmjgh-11-4-s002.pdf]

Supplemental file 2: Standard notation version of process map

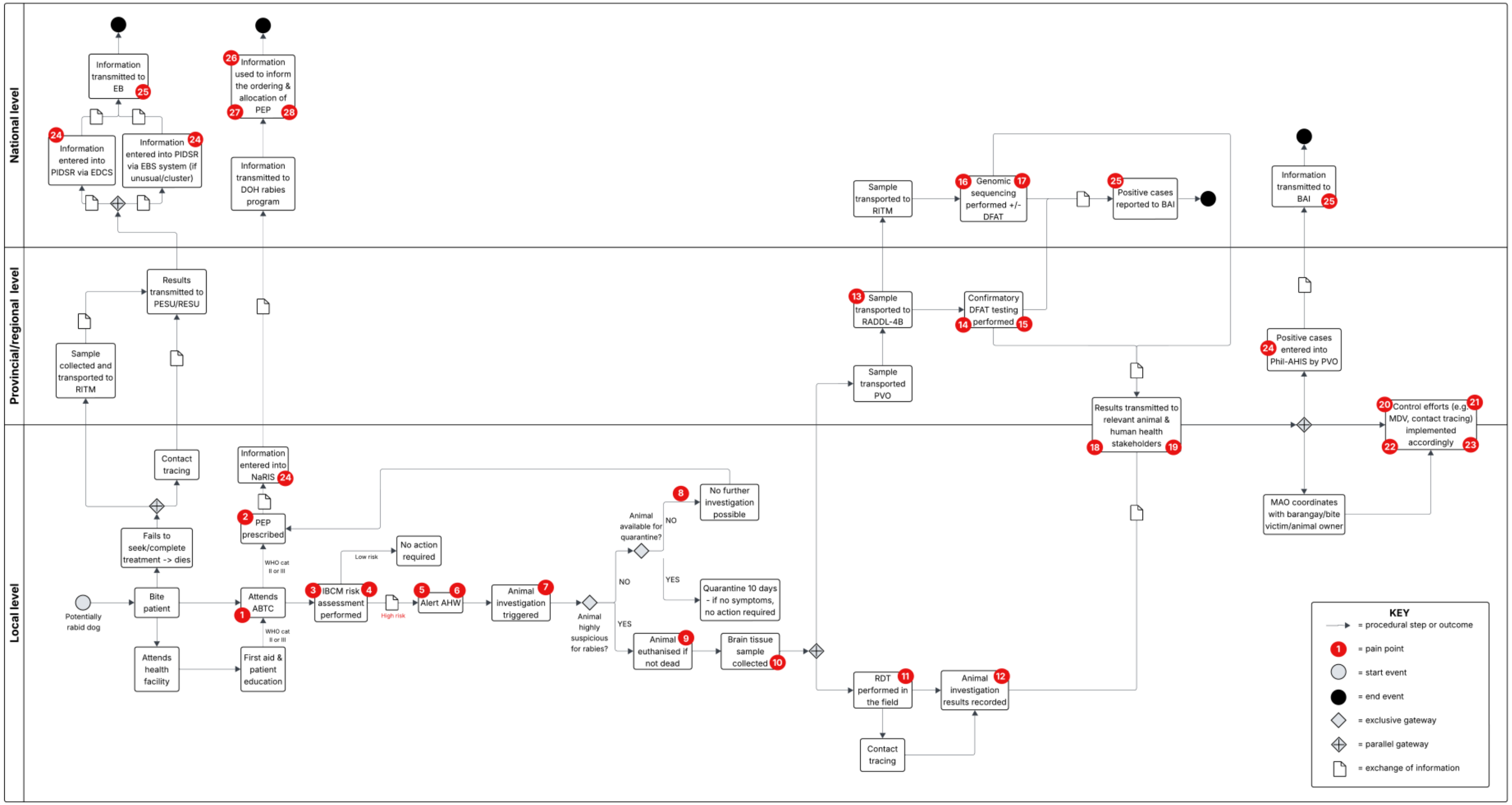

The map is divided into three operational levels: local, provincial/regional and national. Due to the nature of rabies transmission, we focused our map on the steps that unfold after a potentially rabid dog bites a human (red box). However, these processes can also be triggered by the identification and investigation

of a potentially rabid animal, without the involvement of a bite patient. The numbered red circles show pain points identified by stakeholders. Example pain points are described in Table 1, and the full list of pain points is in Supplementary File 3.

Acronyms: ABTC, Animal Bite Treatment Centre; AHW, Animal Health Worker; BAI, Bureau of Animal Industry (Department of Agriculture); DFAT, Direct Fluorescent Antibody Test; DOH, Department of Health; EB, Epidemiology Bureau (DOH); EBS, Event-Based Surveillance; EDCS, Epidemic-prone Disease Case Surveillance; IBCM, Integrated Bite Case Management; MAO, Municipal Agriculture Office; MDV, Mass Dog Vaccination; NaRIS, National Rabies Information System; PEP, Post-Exposure Prophylaxis; PESU/RESU, Provincial/Regional Epidemiology and Surveillance Unit; Phil-AHIS, Philippine Animal Health Information System; PIDSR, Philippine Integrated Disease Surveillance and Response; PVO, Provincial Veterinary Office; RADDL, Regional Animal Disease Diagnostic Laboratory; RDT, Rapid Diagnostic Test; RITM, Research Institute for Tropical Medicine; WHO cat. II/III, World Health Organization exposure categories II and III.
