# Supplementary material for: Process mapping the One Health response to a rabies outbreak in the Philippines
Source: BMJ Glob Health. 2026 Apr 2;11(4):e020482. doi: 10.1136/bmjgh-2025-020482 (PMC13052803; doi:10.1136/bmjgh-2025-020482)
Supplement: online supplemental file 3 [file bmjgh-11-4-s003.pdf]

**Supplemental file 3: Finalised list of pain points**

These pain points correspond to those shown on the process maps (Figure 3 and Supplementary File 2). The yellow highlighted text represents edits made by participants during the workshop and the coloured backgrounds correspond to how the map was divided up for use by different stakeholder groups during the workshop (see Figure 3).

1. Failure to present to ABTC: lack of awareness and/or logistical/financial/cultural barriers; healer (tandok) does not refer victim to ABTC; negative perception of PEP availability and/or hospital experience.
2. Patients fail to complete PEP: lack of awareness and/or logistical/financial/cultural barriers; poor PEP availability.
3. PHW confusion regarding what constitutes a "high risk" bite according to IBCM risk assessment – affects triggers for animal investigation and resulting surveillance data.
4. Risk assessment results not recorded/delayed: lack of time and/or human resources; app/technological challenges.
5. PHWs fail to alert AHWs (or SPEEDIER team) and/or RESU to high-risk case (or do so after a significant delay): lack of time/human resources/understanding of importance.
6. AHWs receive incomplete risk assessment information from PHWs (e.g., lacking patient details) required for timely investigation: lack of resources (e.g. forms); uncooperative patient/relatives; communication/language barrier.
7. Animal investigations not carried out: AHWs not alerted by PHWs/alerted too late; AHWs lack time/resources to investigate; investigations not considered useful/prioritised given other duties; pet owners directly go to MAO to request biting animal to be tested; or other reasons?
8. Animal missing/consumed/decomposed. AHW cannot sample or investigate further.
9. Safe euthanasia difficult: MAOs not legally allowed to euthanise animals; police refusal to assist (in shooting potentially rabid animals); crowd control; public backlash etc.
10. AHWs unwilling/unable to collect sample: lack confidence; lack time/human resources; lack of training; health and safety concerns; lack PPE/equipment/transport/access to carcass; fear poor sample quality due to decay, sampling process unpleasant; other reasons?
11. RDTs rarely performed (see 9): lack access to RDTs; lack of training (or ineffective training); legality of releasing unofficial results? This significantly extends sample results turnaround time.
12. Animal investigation results not recorded: lack of human resources/understanding of importance; the need to complete multiple RADDL forms if multiple bite victims.

13. Delay in sample reaching lab from PVO: organisational; logistical (e.g., limited ferries); lack of human resources (e.g., only 1 person at PVO responsible for dispatch – often absent/busy); lack of other resources (e.g., Styrofoam boxes used to transport samples).

14. At time of interviewing, RADDL microscope broken* (RDT backup only). Samples had to be be transported to RITM for dFAT confirmation – requires human resources (hand delivered samples), logistically difficult and expensive (cost falls to PVO).

15. RADDL concerns over release of RDT results (risk license will be revoked), delay while waiting for dFAT results.

16. Delays in releasing results: institutional bureaucratic processes: late arrival after transport from RADDL, VRD open weekday office hours only and transport still required to SPL for processing. SPL open 24/7, yet VRD signoff required for results release. Other issues include: sample sometimes lack submission form (only a label) with information needed for results form to be released; no protocol for reporting if DFAT negative.

17. Test result report forms contain inaccurate information: recent change in database for incoming samples leading to incorrect data entry**.

18. Investigation and laboratory results not communicated to PHWs and other parties: oversight; lack of established intersectoral communication; lack of understanding of importance; uncertainty regarding who requires information.

19. Multiple sample IDs: different conventions (MAO/PVO, RADDL, RITM and SPEEDIER) complicates analysis and communication.

20. Animal and public health responses not synchronised in terms of measures taken and resources mobilised.

21. Turnaround time between sample collection and result transmission too long to allow community to be alerted in a timely manner and response measures to be implemented swiftly e.g., dog vaccination, community education re rabies risk/the need to seek PEP in the event of a bite, enforcement of responsible pet ownership ordinances.

22. Implementation of response measures hampered: lack of material/human resources/funding (difficult to convince mayors to allocate budget for rabies (vaccines, etc)); bureaucratic barriers. Dog vaccination limited by lack of vaccines. Recent Mandanas ruling has exacerbated this issue.

23. Confusion and reluctancy surrounding declaring an outbreak: what constitutes an outbreak; what is the procedure for declaring an outbreak (to allow municipalities/province to apply for emergency funds); potential negative consequences for the province?

24. Roles of different stakeholders (especially those at a regional/national levels) not clearly defined in the event of an outbreak.

25. Duplicated/inconsistent data entry due to NaRIS backlog and poor functionality, unreliable internet connectivity.

26. Inaction of high-level stakeholders (e.g., National Rabies Program): data underutilised; lack of feedback (e.g., BAI failing to send vaccine bank request to WOAH).

27. NaRIS records unreliable; PEP supply needs must be extracted from manual reports.

28. Bulk of the rabies budget spent on RIG.

29. Bidding process to obtain PEP vaccines is poorly designed and time consuming
