# Supplementary material for: Process mapping the One Health response to a rabies outbreak in the Philippines
Source: BMJ Glob Health. 2026 Apr 2;11(4):e020482. doi: 10.1136/bmjgh-2025-020482 (PMC13052803; doi:10.1136/bmjgh-2025-020482)
Supplement: online supplemental file 4 [file bmjgh-11-4-s004.pdf]

Supplemental file 4: Process mapping workshop data capture spreadsheet

Data collected by our facilitators during our stakeholder workshop held in Romblon on 10th August 2023.

Regional and national-level stakeholder group

Cluster	Process	Pain Points<br>*(Session 1: ask participants to use sticky notes or pentel pen + chat from online participants)*	Revised Pain Points<br>(Session 2)	Actionable solutions<br>(Session 3)	Responsible Authority (Person/Organization)	Timeframe	Indicators	Comments
Regional/National	Results transmitted to PHWs + provincial stakeholders	Investigation and laboratory results not communicated to PHWs and other parties: oversight; lack of established intersectoral communication; lack of understanding of importance; uncertainty regarding who requires information.	Investigation and laboratory results not communicated to PHWs and other parties: oversight; lack of established intersectoral communication; lack of understanding of importance; uncertainty regarding who requires information.	RHU and other health units should be copied in releasing of confirmatory results (for early warning, contact tracing); ProVet/MAO should also share results with public health counterparts;				
				Develop a handbook and refer back to national rabies MOP - outlining clear delineation of functions and responsibilities of each office involved in sample submissions; RITM has outbreak sample submission manual				
Regional/National	Results transmitted to PHWs + provincial stakeholders	Multiple sample IDs: different conventions (MAO/PVO, RADDL, RITM and SPEEDIER) complicates analysis and communication.	Multiple sample IDs: different conventions (MAO/PVO, RADDL, RITM and SPEEDIER) complicates analysis and communication.	Once RADDL receives a sample for testing, they can immediately share the accession number with SPEEDIER, to avoid confusion in following up				
Regional/National	Results transmitted to PHWs + provincial stakeholders	AH and PH response NOT synchronized in terms of mobilization of resources, vaccines, funds and response operations, coordination, shared resources	AH and PH response NOT synchronized in terms of mobilization of resources, vaccines, funds and response operations, coordination, shared resources	Reactivate the Provincial and Municipal Rabies Committees; anchored in rabies laws and ordinances				
Regional/National	Control efforts implemented accordingly	Turnaround time between sample collection and result transmission too long to allow community to be alerted in a timely manner and response measures to be implemented swiftly e.g., dog vaccination, community education re rabies risk/the need to seek PEP in the event of a bite, enforcement of responsible pet ownership ordinances.	Turnaround time between sample collection and result transmission too long to allow community to be alerted in a timely manner and response measures to be implemented swiftly e.g., dog vaccination, community education re rabies risk/the need to seek PEP in the event of a bite, enforcement of responsible pet ownership ordinances.	Use of system of any infectious disease sample (COVID, polio, measles, etc) to RITM through courier partner (JRS), direct from province to RITM but information is still shared to all channels (RESU to RITM)				
Regional/National	Control efforts implemented accordingly	Implementation of response measures hampered: lack of material/human resources/funding; bureaucratic barriers. Dog vaccination limited by lack of vaccines. Recent Mandanas ruling has exacerbated this issue.	Implementation of response measures hampered: lack of material/human resources/funding; bureaucratic barriers. Dog vaccination limited by lack of vaccines. Recent Mandanas ruling has exacerbated this issue; mayors hard to convince to allocate budget for rabies (vaccines, etc)					
Regional/National	Control efforts implemented accordingly	Confusion and reluctancy surrounding declaring an outbreak: what constitutes an outbreak; what is the procedure for declaring an outbreak (to allow municipalities/province to apply for emergency funds); potential negative consequences for the province?	Confusion and reluctancy surrounding declaring an outbreak: what constitutes an outbreak; what is the procedure for declaring an outbreak (to allow municipalities/province to apply for emergency funds); potential negative consequences for the province?	PDOHO provides technical assistance, including providing information regarding declaration of outbreaks (based on existing laws, RA 11332, Rabies MOP); already included in orientations with MESU and HESU; advocate to LCEs the positive effectives of declaration of outbreak such as access to funds for response				
Regional/National	Control efforts implemented accordingly	Roles of different stakeholders (especially those at a regional/national levels) not clearly defined in the event of an outbreak.	Roles of different stakeholders (especially those at a regional/national levels) not clearly defined in the event of an outbreak.					
Regional/National	Positive cases entered into Phil-AHIS by PVO, Information entered into PIDSR, EBSS, NARIS	Duplicated/inconsistent data entry.	Duplicated/inconsistent data entry; NaRIS backlog and glitches, challenged by internet connectivity	Designated and dedicated encoder for NaRIS				
Regional/National	Information transmitted to DA, EB, DOH, Positive cases reported to BAI	Inaction of high-level stakeholders (e.g., National Rabies Program): data underutilised; lack of feedback (e.g., BAI failing to send vaccine bank request to WOAH).	Inaction of high-level stakeholders (e.g., National Rabies Program): data underutilised; lack of feedback (e.g., BAI failing to send vaccine bank request to WOAH).					
Regional/National	Information used to inform and ordering and allocation of PEP	NaRIS records unreliable; PEP supply needs must be extracted from manual reports.	NaRIS records unreliable; PEP supply needs must be extracted from manual reports.					
Regional/National	Information used to inform and ordering and allocation of PEP	Bulk of the rabies budget spent on RIG.	Bulk of the rabies budget spent on RIG.					
Regional/National	Information used to inform and order[illegible] and allocation of PEP	Bidding process to obtain PEP vaccines is poorly designed and time consuming	Bidding process to obtain PEP vaccines is poorly designed and time consuming					

Human health stakeholder group

Cluster	Process	Pain Points<br>*(Session 1: ask participants to use sticky notes or pentel pen + chat from online participants)*	Revised Pain Points<br>(Session 2)	Actionable solutions<br>(Session 3)	Responsible Authority (Person/Organization)	Timeframe	Indicators	Comments
**Human Health**	Attends ABTC	Failure to present to ABTC: lack of awareness and/or logistical/financial barriers; healer (tandok) does not refer victim to ABTC .	Failure to present to ABTC:					
			Lack of awareness of the patient on the importance of rabbies deasese and responsible pet ownership	Intensification of information and education campaign, through distribution of IEC materials, conduct of rabbies lecturing FDS, Barangay assemblies, attendance to health boards, rabbies awareness month, use of multimedia, integration of rabbies dse in school curriculum.	RHU, Municipal link, barangay officials, school nurse, MAO ABTC hospitals	From August to December 2023	90 % of animal bite cases avail ABTC services or consulted to RHU	Request from external support from DOH, SPEEDIER and other organizations.
			Cultural belief (religion), Strong belief to tandok, use of tradittional healing provess like snake stone, garlic,	Encourage tandoks to be part of team of ABTC's / resolution to penalize traditional rabbies healer	SB (Municipal Council) on health	From August to December 2023	Passage of Rabbies ordinance penalizing tandok/ traditional healers	
			Logistical and financial barriers					
			Lack of vaccine available	Ensure availability and regular procurement of rabbies vaccine annualy. Sharing of anti rabbies vaccine from LGU's without ABTC	MHO	Jan to December 2024	Available vaccine at all times	
			Poor refferal system	Strengten the refferal system through provisions of two way refferal form and increase and awareness health facilty of the approved animal bite refferal system.	RHU and ABTC, Hospital	From August to December 2023	Functional two way animal bite referral system	
			Fear of procedural barrier in the facility such as: reequirement for antigen testing, long line of patients, healthcare personnel attitude	Removed antigen request as per case basis, improve facility patient turn around time and establish inter personnal communication skills	RHU and ABTC, Hospital	From August to December 2023	Less complaint from client satisfation survey form	
			Decieving about the decease condition	Councelling of patient	RHU, ABTC, Hospital	From August to December 2023	100% of ABTC patient underwent counselled	
	1A. RESU surveillance	Failure to report to provincial surveillance	Failure to report to provincial surveillance:					
			Data not complete / lack of time/ lack of human resources/patient conditionNo designated DSO on private hospital,	Hired / designate DSO	RHU, ABTC, Hospitals	From August to December 2023	1 DSO per facility	
			lack of training	Request for training and updating	RHU, ABTC, Hospitals	From August to December 2023	DSO trained	
**Human Health**	PEP Prescribed	Patients fail to complete PEP: logistical and/or financial barriers.	Patients fail to complete PEP:					
			FInancial barrier	Provide financial assistance from LGU ang responsibility of the owner of the biting animal	RHU	From August to December 2023	Provided financial assistance	
			Cultural beliefs	Conduct rabbies campaign	RHU, ABTC, Hospital	From August to December 2023	Complete PEP	
			Logistical barrier: Vaccine availability	Ensure availability and regular procurement of rabbies vaccine annualy. Sharing of anti rabbies vaccine from LGU's without ABTC	MHO	Jan to December 2024	Available vaccine at all times	
			Conflict of schedule with regards to vaccination schedule.Lack of communication on the schedule of ABTC, ABTC not open 24 hrs. (mon-Friday), availability of healthcare pesrsonnel and vaccine,	Issue advisory on the schedule of ABTC, inform all stakeholders and nearby reffering facility	RHU, ABTC, Hospital	From August to December 2023	Complete PEP	
			Fear of adverse effect of vaccine	Conduct awareness of AEFI	RHU, ABTC, Hospital	From August to December 2023	Complete PEP	
			Lack of awareness with the vaccine.					
			Loss of vaccination card	Follow up and provide replacement of vaccination card,				
			Believe that if the animal is ok they dont have to finish the vaccination.	Increase awareness				
			Lack of awarenes on vaccine updates and management of PHN.	Training ABTC staff				
Human Health	IBCM Risk assessment performed	PHW confusion regarding what constitutes a "high risk" bite according to IBCM risk assessment – affects triggers for animal investigation and resulting surveillance data.	PHW confusion regarding what constitutes a "high risk" bite according to IBCM risk assessment – affects triggers for animal investigation and resulting surveillance data.	Orientation of updated IBCM				
			Lack of orientation of HCW.					
			IBCM					
			No permanent staff in ABTC	Provide RHU staff				
			No DSA	employ DSA				
**Human Health**	IBCM Risk assessment performed	Risk assessment results not recorded/delayed: lack of time and/or human resources; app/technological challenges.	Risk assessment results not recorded/delayed: lack of time and/or human resources; app/technological challenges.	Re orientation on IBCM and App	RHU, ABTC, Hospital	From August to December 2023	Oriented ABTC staff on updated IBCM and use of App.	
			Lack of orientation on the new app.					
			Lack of time / under staff/ multi tasking / human resources	Time management and hiring of additional in the ABTC				
Human Health	Alert AHW	PHWs fail to alert AHWs (or SPEEDIER team) to high-risk case (or do so after a significant delay): lack of time/human resources/understanding of importance.	PHWs fail to alert AHWs (or SPEEDIER team) to high-risk case (or do so after a significant delay): lack of time/human resources/understanding of importance.	Reorientation of IBCM	RHU, ABTC, Hospital	From August to December 2023	All high risk cases reported to IBCM and encoded to app.	
			Not part of process (RHU to MAO coordination)	Idintification and dilination of ABTC and MAO				
			Lack of communication / details	Improve communication				
			Not practice of ABTC to contact MAO of other LGU	Completion of data				
			Incomplete data					
			Not aware on the process					
			No direct communication of AHW and RHU.	Use of SPEEDIER app				
Human Health	Alert AHW	AHWs receive incomplete risk assessment information from PHWs (e.g., lacking patient details) required for timely investigation.	AHWs receive incomplete risk assessment information from PHWs (e.g., lacking patient details) required for timely investigation.	Complete the IBCM risk assessment form	RHU, ABTC, Hospital	From August to December 2023	100% of animal bite cases and conducted risk assessment and encoded to App	
			Late information					
			Uncooperative relatives / patient.					
			Availability of proper forms (logistics)	Reproductuion of forms				
			Travel	Provision of travel				
			Communication / language barrier	Localization of IEC materials to local dialect				

Animal health stakeholder group

Cluster	Process	Pain Points<br>*(Session 1: ask participants to use sticky notes or pentel pen + chat from online participants)*	Revised Pain Points<br>(Session 2)	Actionable solutions<br>(Session 3)	Responsible Authority<br>(Person/Organization)	Timeframe	Indicators	Comments
Animal Health/Laboratory	Animal investigation triggered	Animal investigations not carried out AHWs not alerted by PHWs/alerted too late; AHWs lack time/resources to investigate; investigations not considered useful/prioritised given other duties; or other reasons?	Pet owners directly go to MAO Office to request biting animal to be tested.Animal investigations not carried out AHWs not alerted by PHWs/alerted too late; AHWs lack time/resources to investigate; investigations not considered useful/prioritised given other duties; or other reasons?	**SOlution for high-risk and low-risk bite cases:** If Patient approaches RHU first (with oABTC) he should be advised by RHU to MAO counterpart. If patient approaches MAO first, MAO will endorse to RHU. For municipalities without ABTC whose destination is a provincial hospital (RPH or Don Modesto), the RPH or DM or any ABTC should contact the MAO of residence of patient<br>**Timeframe:** 1 month<br>**Indicator: recording of information.** Number of reported case. Records of MAO and RHU should tally. If 10 high-risk bite cases referred by RHU without ABTC, MAO should also have 10 animal investigations. Use log book. Use RADDL form.				
Animal Health/Laboratory	Animal missing/consumed/decomposed	Animal missing/consumed/decomposed.<br>AHW cannot sample or investigate further.	"Animal missing/consumed/decomposed.<br>AHW cannot sample or investigate further."	**Solution:** advise precautionary measures to affected area (keep your dog tied or indoors rule all the time), 30 day observation period but not lockdown, and health awareness campagin (coordinate with baranggay - use assigned village councilor to do bandillo campaign)<br>**Timeframe:** 14 days<br>**Indicator:** Number of reported of dFAT positive or negative cases from RADDL				
	Animal euthanised if not already dead	Safe euthanasia difficult: AHWs lack necessary equipment; crowd control; police refusal; public backlash etc.	Requires licensed veterinaraian.Euthanasia not yet utilized due to lack of trained staff and issue about animal welfare.We just wait to animal to die.	**Solution:** since no budget for purchasing of euthanizing agent and no license vet to perform euthanasia, ask owner to sacrifice animal or wait for natural death of suspected rabid animal. make waiver form to release liability sa MAO directed to pet owner. *Kasunduan sa pagsuko ng aso* (Agreement to surrender the dog) to be distributed to all MAOs. euthanize only when animal is threat to human life.<br>**Timeframe:** 30 days<br>**Indicator:** increase in new cases				
Animal Health/Laboratory	Brain tissue sample collected	AHWs unwilling/unable to collect sample: lack confidence; lack time/human resources; health and safety concerns; lack PPE/equipment/transport/access to carcass; fear poor sample quality due to decay, sampling process unpleasant; other reasons?	Untrained MAO Staff refused to collect brain tissue.Prefer the whole head as prescribed by RADDL<br>Established second inline personnel to collect sample in absence of the MAO	**Solution:** as of now since there is no training, we will submit head. MAO will establish second inl ine for rabies sample collection. And both first and second in line must be trained.<br>**Timeframe: 3 months**<br>**Indicator:** 2 trianing certificates per MAO office				
Animal Health/Laboratory	RDT performed in the field	RDTs rarely performed (see 9): lack access to RDTs; legality of releasing unofficial results? This significantly extends sample results turnaround time.	Untrained MAO Staff refused to perform RDT,insufficient RDT Kits and personnel were not confident with online training	**Solution: While untrained,** PVO will perform RDT then give back results down to MAO. If RDT- it is better to be prioritized for dFAT. After training, MAO will do RDT on the field.<br>**Timeframe:** 1 month<br>**Indicator: number of RDTs consumed, number of results of RDTs**				
Animal Health/Laboratory	Animal investigation results recorded	Animal investigation results not recorded:<br>lack of human resources/understanding of importance.	The need to accomplish multiple RADDL forms if multiple bite victims are present	**Solution:** 1 RADDL submission form per biting animal, even if multiple bite victims. Write at the back of first page. To prevent confusion and writin gtoo many forms. And complete RADDL submission form - all fields<br>Timeframe: 1 month<br>Indicator: complete details/incomplete report from RADDL				
Animal Health/Laboratory	Sample transmitted to RADDL	Delay in sample reaching lab from PVO:<br>organisational; logistical (e.g., limited ferries); resource reasons (e.g., only 1 person at PVO responsible for dispatch – often absent/busy).	Delay in sample reaching lab from PVO:<br>Unavailability of storage box/styrobox on MAO Office organisational; logistical (e.g., limited ferries); resource reasons (e.g., only 1 person at PVO responsible for dispatch – often absent/busy).	**Solution:** request allocation of supplie (styrofoam box budget) from mayor for surveillance purposes - to include in MOOE<br>**Timeframe:** 12 months<br>Indicator: reports from MAOs regarding styros purchased	MOOE - Memorandum of Operational Expenses			
Animal Health/Laboratory	Confirmatory dFAT testing performed	At time of interviewing, RADDL microscope broken* (RDT backup only). Samples had to be be transported to RITM for dFAT<br>confirmation – requires human resources (hand delivered samples), logistically<br>difficult and expensive (cost falls to PVO).	At time of interviewing, RADDL microscope broken* (RDT backup only). Samples had to be be transported to RITM for dFAT<br>confirmation – requires human resources (hand delivered samples), logistically<br>difficult and expensive (cost falls to PVO).	**Solution:** Regardless of dFAT +/- forward lab result to counterpart ABTC nurse, RHU/MHO (who will inform the bite patient), concerned LGU. If no bite victim, report is for MAO, and concerned brgy only.<br>**Timeframe:** 1 month<br>**Indicator:** reports of RHU/MHO of not receiving dFAT + or - .				
Animal Health/Laboratory	Confirmatory dFAT testing performed	RADDL concerns over release of RDT results<br>(risk license will be revoked), delay while waiting for dFAT results.						
Animal Health/Laboratory	Genomic sequencing performed	Delays in releasing results:<br>institutional bureaucratic processes: late arrival after transport from RADDL, VRD open weekday office hours only and transport still required to SPL for processing. SPL open 24/7, yet VRD signoff required for results release. Other issues include: sample sometimes lack submission form (only a label) with information needed for results form to be released.	Results were not relayed to respective facilities concerned(RHU,ABTC Nurse and LGUs especially when the results turned negative dFat. No protocol on reporting if dfat neg	**Solution:** Regardless of dFAT +/- forward lab result to counterpart ABTC nurse, RHU/MHO (who will inform the bite patient), concerned LGU. If no bite victim, report is for MAO, and concerned brgy only.<br>**Timeframe:** 1 month<br>**Indicator:** reports of RHU/MHO of not receiving dFAT + or - .				
Animal Health/Laboratory	Genomic sequencing performed	Test result report forms contain inaccurate information: recent change in database for incoming samples leading to incorrect data entry**.						
