# Supplementary material for: Process mapping the One Health response to a rabies outbreak in the Philippines
Source: BMJ Glob Health. 2026 Apr 2;11(4):e020482. doi: 10.1136/bmjgh-2025-020482 (PMC13052803; doi:10.1136/bmjgh-2025-020482)
Supplement: online supplemental file 5 [file bmjgh-11-4-s005.pdf]

Supplemental file 5: Executive summary and urgent appeal

A letter sent to the Governor of Romblon Province on 24th August 2023 summarising the findings of our stakeholder workshop and appealing for a rabies outbreak to be officially declared in the province.

24 August 2023

HON. JOSE R. RIANO
Governor
Province of Romblon

Attention: **LINO MARCUS M. VIOLA III, MD, DPCR, MBA**
OIC - Provincial Health Officer II

PAUL MIŇANO, DVM
Provincial Veterinarian

Subject: Urgent Appeal for Tablas Island Rabies Outbreak Response

Dear Honorable Governor Riano,

Rabies kills >200 people annually in the Philippines. Prior to 2020, Romblon Province in Region IV-B MIMAROPA was rabies-free, but animal cases have been detected on Tablas Island since late 2022. This year, two human rabies deaths have been confirmed. Neither victim sought po-exposure prophylaxis from Romblon's four Animal Bite Treatment Centers following dog bites in December 2022 and January 2023. As of 23 August 2023, 38 dogs have tested rabies positive at the Regional Animal Disease Diagnostic Laboratory (RADDL IV-B) covering nearly all municipalities on Tablas Island (please refer to Annex).

On 10 August 2023, a workshop involving stakeholders from all nine municipalities in Tablas was organized by the SPEEDIER Team, your local health development partner for enhancing disease surveillance for rabies elimination. Participants included 3 Municipal Health Officers, 4 Municipal Agriculturists together with the Rabies Coordinator from the Provincial Veterinary Office, the Provincial Health Office and Disease Surveillance Officers from the Provincial Department of Health Office. Delegates from regional and national stakeholders from the Center for Health Development (CHD-MIMAROPA), RADDL IV-B, and the Research Institute for Tropical Medicine joined the proceedings virtually. The workshop was conducted to map the processes of the outbreak response so far; identify challenges and successes within the response; and work towards actionable solutions to the challenges identified.

The participants universally agreed on the urgent need to improve the response, coordinate actions and overcome immediate practical challenges:

- Individual response efforts have been commendable, but lack of communication and coordination between and within sectors and across LGUs has led to a fragmented ineffective response.

- Failure to officially declare the outbreak has meant communities are not sensitized to the heightened risk, dangers of dog bites and importance of postexposure vaccination or the need to report suspicious animals.
- Intersectoral communications have improved, but limited sharing of reports still hampers coordination.
- The response has been most constrained by inadequate budget. The critically short supply of dog vaccines is prolonging the outbreak, putting lives at risk and is costly for the longer term.

High dog vaccination coverage across all municipalities can restore rabies freedom. A concerted One Health response would be an exemplar to other regions of the Philippines similarly facing rabies challenges and would be in line with the ***One Romblon*** vision. **Stakeholders call upon decision-makers to recognize the continuing Tablas rabies outbreak and recommend to the Governor as Chair of the Provincial Rabies Committee to mobilize resources for an island-wide coordinated response.**

We convey this urgent message on behalf of our partners and stakeholders in Romblon.

Yours respectfully,

Mary Elizabeth Miranda
SPEEDIER Team Leader

ANNEX

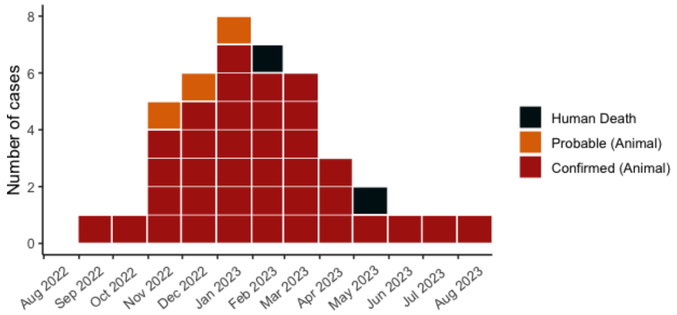

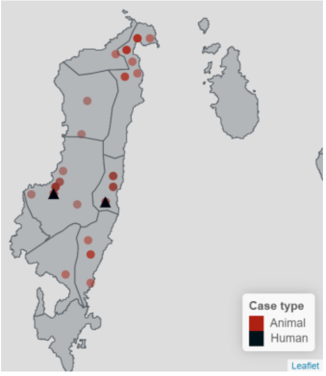

Figure 1. Epidemic curve of rabies cases and deaths on Tablas island from the last year. Further details: http://boydorr.gla.ac.uk/rabies/SPEEDIER/
