# Supplementary material for: Process mapping the One Health response to a rabies outbreak in the Philippines
Source: BMJ Glob Health. 2026 Apr 2;11(4):e020482. doi: 10.1136/bmjgh-2025-020482 (PMC13052803; doi:10.1136/bmjgh-2025-020482)
Supplement: online supplemental file 6 [file bmjgh-11-4-s006.pdf]

Supplemental file 6: Summary of findings and recommendations

A document sent to all workshop participants and local, regional and national stakeholders summarising the findings of our workshop and outlining our recommendations.

Process Mapping Workshop: Summary of Findings and Recommendations

Background

Rabies kills >200 people annually in the Philippines. Prior to 2020, Romblon Province in Region IV-B MIMAROPA was rabies-free. However, since October 2022, 52 confirmed animal cases have been detected on Tablas Island, covering nearly all municipalities. So far in 2023, three human rabies deaths have also been confirmed. None of the victims sought post-exposure prophylaxis (PEP) from Romblon's four Animal Bite Treatment Centers (ABTCs).

On 10th August 2023, the SPEEDIER team organized a workshop with stakeholders from all nine municipalities on Tablas Island. The workshop used a process mapping approach to establish a clear picture of the outbreak response so far; identify challenges and successes within the response; and work towards actionable solutions to challenges identified.

The following findings and recommendations are the output of this workshop, based on both stakeholder input and SPEEDIER technical contributions.

Key findings

- Individual response efforts have been commendable but insufficient collaboration between and within sectors and across LGUs has led to a fragmented ineffective response. One Health communication has improved in certain contexts, but limited sharing of case reports still hampers coordination.
- Failure to officially declare the outbreak has meant communities remain unsensitized to the heightened risk, importance of seeking post-exposure prophylaxis in the event of a bite, or the grave need to report suspicious animals.
- The process of testing samples from potentially rabid animals is currently inefficient and affected by multiple bottlenecks. The turnaround time is often too long to allow timely alerts to affected communities and swift implementation of response measures.
- The response has been most constrained by inadequate budget. The critically short supply of dog vaccines is prolonging the outbreak, putting lives at risk and will prove costly in the longer term.
- Critical data gathered from surveillance activities are often not submitted to appropriate disease reporting channels due to overburdened personnel or are not acted on by relevant regional or national authorities.

Our recommendations address the challenges outlined in these key findings. They comprise a range of suggested improvements, including tangible actions that can be implemented quickly by local stakeholders on a municipal or provincial level, and more structural changes requiring regional or national level action/ investment. We understand that implementing higher-level changes will take time and be logistically challenging, but we hope that this document can serve as a useful resource for stakeholders advocating for those changes. SPEEDIER can provide guidance as needed when it comes to implementing these recommendations and will continue to provide support with enhancing rabies surveillance in Romblon, Oriental Mindoro and Marinduque.

Key recommendations

Short-term (priority) objectives

1. Officially declare a rabies outbreak in the province of Romblon.
2. Coordinate animal and human health response efforts in terms of operations and resources.
3. Intensify education campaigns to raise awareness of the current outbreak.

Medium-term objectives

1. Ensure correct identification and detailed recording of bites by animals with signs indicative of rabies (i.e., “high risk” bites).
2. Ensure consistent communication of "high risk" bites to Municipal Agricultural Officers (MAOs) by ABTC or RHU staff.
3. Ensure consistent and timely investigation and comprehensive recording of all “high risk” biting animals.
4. Expedite animal sample referral, transport and processing for confirmatory diagnosis.
5. Update test result dissemination protocols.
6. Ensure rabies case data received by regional and national animal and human health bodies is reviewed promptly and acted upon appropriately.
7. Refine and enforce reporting protocols to public health and animal health disease prevention and control programs reinforcing One Health systems approaches.

Long-term objectives

1. Strengthen the patient and specimen referral processes for both human and animal health systems.
2. Address barriers to post-exposure prophylaxis completion.
3. Further develop the existing national guidance on rabies outbreak management and build communities of practice to achieve the 7-1-7 target for general outbreak detection and response.

For more detailed recommendations please see the attached appendix.

If you have any questions, or if SPEEDIER can provide any guidance or support when it comes to implementing these recommendations, you can reach out to us through **info@fetpafi.org**.

Appendix

Priorities coloured by time scale: **!!! short-term**; **!! medium-term**; and **! long-term**

Table 1: Coordination and response

Action point	Responsible agency	Notes
**!!! Declare a rabies outbreak in the province of Romblon.**	Provincial Governor (as head of the Provincial Rabies Committee)	*The existence of **functional** surveillance units at all levels (municipal, provincial, regional) is essential for timely detection and declaration of outbreaks.*<br>*Declaration is of vital importance if:*<br>*1. An outbreak is in a rabies-free area. 2. The outbreak affects at least one municipality already (animal / human cases). 3. Declaration will facilitate access to material and political support to tackle the outbreak.*<br>*4. Declaration will increase awareness about heightened risks & importance of responding appropriately to bites /potential rabid animals.*
**! Further develop existing guidance on rabies outbreak management.** Ensure guidance includes clear rabies-specific answers to these questions:	National Rabies Prevention and Control Committee (NRPCC)	*Further develop the guidance within the NRPCC Manual of Procedures. The guidance should take into consideration the zoonotic nature of rabies and align with pre-existing national and international guidance for management of notifiable diseases (e.g. Republic Act No. 11332, WHO International Health Regulations).*
Q1. What constitutes an outbreak?		*For rabies-free areas, SPEEDIER recommends **any confirmed animal case** (RDT or dFAT) **&/ human case** (clinical or laboratory confirmed) be considered evidence of an outbreak. The time until outbreak detection (and action), directly correlates with the resulting outbreak size and extent[1]. Therefore, fast detection, declaration and response is vital.*
Q2. Who can declare an outbreak?		*When responding to zoonotic disease outbreaks, engagement of both animal and human health sectors is critical and should be considered when deciding who can declare an outbreak. Doing so increases chances that outbreaks are detected whilst limited to the animal population & before substantive spread*
Q3. What is the procedure for declaring an outbreak?		*It should also be clear who has authority to declare an outbreak at each level (municipal, provincial, regional).*
Q4. What are the roles & responsibilities of relevant agencies in an outbreak?		

**!! Refine and enforce reporting protocols.**		*Existing protocols for reporting human rabies cases require better enforcement to ensure consistent and timely reporting. There is insufficient guidance on reporting of animal cases (currently by labs to BAI once a month).*
Determine how and to which agencies animal and human cases should be reported.	Local Epidemiology & Surveillance Units,<br>Epidemiology Bureau (EB),<br>Bureau of Animal Industry (BAI),<br>Regional Animal Disease Diagnostic Laboratory IV-B (RADDL IV-B),<br>Research Institute for Tropical Medicine (RITM)	*It is important to take a One Health approach when responding to outbreaks of zoonotic diseases and to ensure the engagement and intersectoral communication of both animal and human health sectors.*
Enforce the consistent reporting of animal and human cases.	Local Epidemiology & Surveillance Units<br>EB<br>BAI<br>RADDL IV-B<br>RITM	*Rabies is considered immediately notifiable by the Philippine Integrated Disease Surveillance and Response (PIDSR) so enforcement of timely consistent case reporting is vital.*
Consider whether currently existing reporting processes can be streamlined to reduce burden on staff.	NRPCC<br>PESU<br>EB<br>SPEEDIER	*Partially addressed during SPEEDIER IBCM refresher training, October 2023. Human health workers were instructed to report only high-risk bite cases to their animal health counterparts moving forward. However, the issue still warrants further attention.*
Train staff to ensure they follow reporting protocols consistently and correctly.	Provincial Veterinary and Agriculture Offices (PVO & PAO)<br>RADDL IV-B<br>RITM<br>SPEEDIER	*Addressed during SPEEDIER IBCM refresher training, October 2023. Reporting protocols were clarified and the importance of following them emphasized. However, the issue still warrants further attention.*
**!! Ensure rabies case data received by regional and national animal and human health bodies is reviewed promptly and acted upon appropriately.**	BAI<br>EB	*Case data should be used to inform policy and practice, and determine which areas may require additional support.*<br>*Data can support an application to the WOAH emergency vaccine bank. Dog vaccines from the vaccine bank could control and prevent outbreaks.*

**!!! Coordinate animal & human health response efforts in terms of operations and resources.**		
Reactivate the Provincial and Municipal Rabies Committees.	Provincial Governor	*This will allow for a proactive approach to rabies control and facilitate the sharing of resources and expertise between sectors.*
Examine budgetary issues preventing LGUs from accessing enough vaccines for regular comprehensive dog vaccination.	Regional Development Board<br>Department of Interior and<br>Local Government	*Controlling dog rabies through mass dog vaccination substantially reduces human exposures and is the most effective strategy for achieving nationwide rabies elimination.*
Consider re-allocating a portion of local government funds to purchase dog vaccines &/ costs of logistics (if vaccines sourced elsewhere).	Mayor of each municipality	*Surveillance data gathered since outbreak start can be used to justify fund and other resources allocation.*
Regular presence of animal health representative &/ rabies champions at Provincial Health Board meetings	Provincial Health Board	*This will facilitate a One Health approach to health promotion in the province and ensure all stakeholders are kept informed of updates.*

[1]Townsend, S.E., et al. 2013. Surveillance guidelines for disease elimination: a case study of canine rabies. *Comparative Immunology, Microbiology and Infectious Diseases*, 36(3):249-61. doi: 10.1016/j.cimid.2012.10.008.

Table 2: Rabies education and awareness

Action point	Responsible agency	Notes
**!!! Intensify education campaigns to raise awareness of the current outbreak, and reinforce community engagement**		*WHO guidelines on "Risk communication and community engagement" can be used to inform all elements of campaigns.*
Make use of social media, information education campaign (IEC) materials & Rabies Awareness Month.	Health Education &<br>Promotions Office (HEPO)	
Ensure rabies education is incorporated into the school curriculum for all children.	District Superintendents	

	(Dept of Education)	
Give educational presentations at barangay assemblies and Family Development Sessions.	Department of Social Welfare & Development	
Use these strategies to increase awareness of heightened risk, vaccine safety, when to seek PEP, financial assistance for PEP and what to do in the event of a possibly rabid animal.	Agencies listed above	

Table 3: Patient risk assessments and animal Investigations

Action point	Responsible agency	Notes
**!! Ensure correct identification and recording of bites by animals with signs indicative of rabies ("high risk" bites).**		
Train ABTC and RHU staff to carry out these procedures consistently and correctly.	SPEEDIER Officers in charge of rabies at the Regional Health Office MIMAROPA	*Addressed during SPEEDIER IBCM refresher training, October 2023. Issue still warrants attention.*
Produce patient information materials in local dialects explaining the risk assessment information required by health centers in the event of a bite.	*Production & distribution of materials*: Centre for Health Development staff at Regional Health Office (supported by SPEEDIER) *Issuing of materials*: ABTCs and RHUs	
**!! Ensure consistent communication of "high risk" bites to MAOs by ABTC and RHU staff.** Train ABTC and RHU staff to consistently communicate "high risk" bites.	SPEEDIER Officers in charge of rabies at the Regional Health Office MIMAROPA, the DA Regional Field Units RADDL, PVO, PAO	*Addressed during SPEEDIER IBCM refresher training, October 2023. Issue still warrants attention.*
**!! Ensure consistent investigation and comprehensive recording of all "high risk" biting animals.**		

Create protocol clarifying responsible MAO in instances where a bite occurs in a municipality without an ABTC.	SPEEDIER<br>PVO / PAO	*Addressed during SPEEDIER IBCM refresher training, October 2023. Issue still warrants attention.*
Ensure information on the importance of preserving MAO access to a biting/suspect animal included in rabies education and awareness campaigns.	HEPO	
Ensure all MAOs have access to an official waiver form (*Kasunduan sa Pagsuko ng Aso*) relinquishing responsibility of the biting animal from the owner to the MAO.	PVO / PAO	
Train staff to improve confidence in rapid testing.	RADDL IV-B<br>SPEEDIER	*Addressed during SPEEDIER IBCM refresher training, October 2023. Issue still warrants attention.*
MAOs to include supplies (e.g., Styrofoam boxes) needed for sample transport in annual budget proposals.	Mayor of each municipality<br>MAOs	
PVO to perform RDTs (and transmit results back to MAOs) until MAOs are confident in performing them.	PVO<br>SPEEDIER	*Addressed during SPEEDIER IBCM refresher training, October 2023. Issue still warrants attention.*
Clarify legality of sharing and acting on the results of rapid diagnostic tests.	BAI	*The latest evidence strongly demonstrates the reliability of a positive RDT[2] and so control measures should be put in place on receipt of a positive result.*
MAOs to complete one RADDL submission form per biting animal, even if there are multiple bite victims. They should ensure all fields are completed.	RADDL IV-B<br>MAOs<br>SPEEDIER	*Addressed during SPEEDIER IBCM refresher training, October 2023. Issue still warrants attention.*

Table 4: Laboratory testing

Action point	Responsible agency	Notes
**!! Expedite animal sample transport & processing**		

Begin using the already established human sample transport chain (as used for COVID, polio, measles etc.) to transport human rabies samples from province to laboratory.	Centre for Health Development MIMAROPA (CHD-MIMAROPA)<br>Provincial Health Office<br>Provincial Epidemiology &<br>Surveillance Unit (PESU)	
Ensure sufficient PVO personnel trained in laboratory referral, sample packing and dispatch so samples reach the laboratory in reasonable time.	PVO	
Ensure shifts of laboratory staff scheduled to ensure (at least) one staff member always available to process samples.	RADDL IV-B Chief<br>Integrated Laboratories Division chief	
RADDL to use "remarks" field to record the date they receive the sample, instead of altering data entered by the MAO.	RADDL IV-B Chief<br>Integrated Laboratories Division chief	*Addressed during SPEEDIER IBCM refresher training, October 2023. Issue still warrants attention.*
MAOs to include email addresses on sample submission forms for prompt results sharing	MAOs<br>SPEEDIER	*Addressed at SPEEDIER IBCM refresher training, Oct 2023. Issue still warrants attention.*
**!! Update test result dissemination protocols.**<br>Clarify how and to whom results of animal sample testing should be shared, considering possible outcomes (positive vs negative result, human bite victims vs no bite victims).	BAI<br>Provincial Governor (head of Provincial Rabies Committee)	*Addressed at SPEEDIER IBCM refresher training, Oct 2023. Issue still warrants attention. A One Health approach to zoonotic outbreaks is needed, with engagement and intersectoral communication of both animal and human health sectors. The importance of informing communities as well as higher level authorities should be considered for dissemination protocols.*

[2]Mananggit, M.R., et al. 2021. Lateral flow devices for samples collected by straw sampling method for postmortem canine rabies diagnosis. PLOS NTDs 15(12): e0009891. https://doi.org/10.1371/journal.pntd.0009891

Table 5: Rabies prevention (post-exposure prophylaxis)

Action point	Responsible agency

! **Strengthen the ABTC referral process.**	
Create a two-way referral form to streamline the referral process.	PHO<br>CHD-MIMAROPA
Consider working with traditional healers to support PEP referrals for those at risk.	Provincial Governor<br>CHD-MIMAROPA<br>NRPCC
Issue advisory on opening hours of ABTCs and RHUs to all referring health facilities.	PHO<br>CHD-MIMAROPA
! **Address barriers to PEP completion**	
Shorten patient wait times using measures such as increasing staffing at peak hours or streamlining administrative processes.	Department of Health<br>CHD- MIMAROPA
Provide replacement vaccine cards for patients where necessary.	ABTCs
Issue a public advisory on ABTC or RHU opening hours	PHO<br>CHD-MIMAROPA
Ensure regular procurement and consistent availability of human rabies vaccines at ABTCs and RHUs	Local Government Units<br>PHO<br>CHD- MIMAROPA
