# Supplementary material for: Process mapping the One Health response to a rabies outbreak in the Philippines
Source: BMJ Glob Health. 2026 Apr 2;11(4):e020482. doi: 10.1136/bmjgh-2025-020482 (PMC13052803; doi:10.1136/bmjgh-2025-020482)
Supplement: online supplemental file 7 [file bmjgh-11-4-s007.pdf]

Reflexivity statement

1. How does this study address local research and policy priorities?

This study focuses on enhancing the response to an outbreak of rabies in a previously rabies-free province of the Philippines. The Philippines reports one of the highest incidences of human rabies in Asia, historically causing between 200-300 deaths per year with a worrying increase to over 350 deaths each year since 2022. The country has been striving to achieve elimination for over a decade, with significant investment in post-exposure prophylaxis and the creation of the National Rabies Prevention and Control Committee and many rabies specific plans and policies. However, the 'Zero by 30' goal currently remains out of reach, with new outbreaks occurring across the country. Our study helps contribute to improve rabies surveillance and outbreak preparedness in the Philippines.

2. How were local researchers involved in study design?

Local researchers were instrumental in the study design and implementation. The study was conceived during a collaborative research project (the Surveillance integrating Phylogenetics and Epidemiology for Elimination of Disease: Evaluation of Rabies Control in the Philippines [SPEEDIER] project) led by Field Epidemiology Training Program Alumni Foundation, Inc. (FETPAFI) and the University of Glasgow. During conversations it was decided that further research was required into the outbreak and that process mapping would be an appropriate approach. Meetings were then held between UK- and Philippines-based authors to determine exactly how the study should be conducted e.g., which stakeholders should be interviewed, what questions should be asked, where/when/how the workshop should be conducted etc.

3. How has funding been used to support the local research team?

The initial collaboration (the SPEEDIER project), jointly funded through the Newton Fund, UKRI and Philippines Council for Health Research and Development (PCHRD), established the researcher base in the Philippines and provided a platform for two Philippines PhD students (Bautista, now a research lead at the Philippines Research Institute for Tropical Medicine and Yuson who recently completed her PhD). The collaboration has now been running for over seven years and lead to other substantive collaborative grants (UKRI and British Academy) where most funds support researchers in the Philippines, that now span multiple organizations (FETPAFI, RITM, University of Ateneo) and disciplines (social and political science, implementation research, epidemiology and phylogenetics).

4. How are research staff who conducted data collection acknowledged?

All research staff who conducted data collection are listed as authors.

5. Do all members of the research partnership have access to study data?

All members of the partnership have access to study data.

6. How was data used to develop analytical skills within the partnership?

Collaborators in Scotland and the Philippines worked together to analyse workshop findings and use them to create "findings and recommendations" policy briefs that were sent to the provincial and regional governments.

7. How have research partners collaborated in interpreting study data?

Please see above.

8. How were research partners supported to develop writing skills?

University of Glasgow-based professor Katie Hampson conducted a “writeshop” in the Philippines attended by the early career authors (both in Scotland and the Philippines) aimed at enhancing scientific writing skills. Two Filipino co-authors have undertaken PhDs at the University of Glasgow during this period (see above), and have corresponding publications covering other aspects of this interdisciplinary collaboration, supported by supervisors at University of Glasgow and in the Philippines (all listed authors).

9. How will research products be shared to address local needs?

This manuscript will be published as open access. Furthermore, the data we collected were used to produce a series of documents for stakeholder use, including an executive summary (sent to high level provincial stakeholders), a more detailed summary of findings and recommendations (sent to all workshop participants and those identified as “responsible agencies”) and individually tailored letters to each responsible agency highlighting specific actionable solutions identified as within their remit. Research outputs have also used to support local advocacy led by FETPAFI resulting in the acquisition of vaccines and other in-kind support for the outbreak response across the region.

10. How is the leadership, contribution and ownership of this work by LMIC researchers recognised within the authorship?

Dr Miranda is listed as second author (with other FETPAFI team members listed as third, fourth and sixth authors) in recognition of our FETAFI collaborators being instrumental in the organisation and facilitation of the work and dissemination of our findings.

11. How have early career researchers across the partnership been included within the authorship team?

Except for the second author and final three authors (who represent the first author’s supervisory team) all authors would be considered early career researchers. The first author is a PhD student.

12. How has gender balance been addressed within the authorship?

Eight authors are female, and seven authors are male, representing a near-equitable gender balance in authorship. The lead author is female.

13. How has the project contributed to training of LMIC researchers?

All listed authors from a LMIC setting have received training or experience in the use of process mapping the response to a zoonotic disease outbreak. This learning will be valuable for use in future disease outbreak scenarios, including further workshops completed since.

14. How has the project contributed to improvements in local infrastructure?

This project has contributed to enhanced management of human bite victims and potentially rabid dogs within the human and animal healthcare systems in the Philippines.

15. What safeguarding procedures were used to protect local study participants and researchers?

Informed consent and participant anonymity (in all disseminated outputs) were used throughout this study to protect local participants, along with general adherence to ethical guidelines found in 1964 Helsinki Declaration and its later amendments. We seured local ethical approval from the Philippine Research Institute for Tropical Medicine Institutional Review Board (approval number: 2019-025) and from the University of Glasgow.

16. Researcher positionality and potential influence on the study

The first author, a UK-based PhD student with a background in veterinary medicine, medical anthropology, systems mapping and rabies surveillance, recognises that her positionality may have influenced the design, facilitation, and interpretation of this study. As a foreign researcher working within a Philippine context, she occupied a position of both “outsider”—due to differences in nationality, cultural background, and institutional affiliation—and “insider-partner”, given ongoing collaborations with local stakeholders through the SPEEDIER project.

These positional dynamics may have shaped participant responses during interviews and workshops. For example, some stakeholders may have moderated critiques of their own institutions or emphasised particular challenges due to perceptions of the researcher’s affiliation with external academic partners or donors. Equally, the researcher’s lack of lived experience within Philippine health and local government systems may have influenced which issues she identified as salient or how she interpreted stakeholder-described constraints, particularly those relating to political dynamics or cultural practices such as traditional healing.

To mitigate these influences, the research was designed and implemented in close partnership with CHD–MIMAROPA and local collaborators, who provided guidance on culturally appropriate engagement, helped validate emerging interpretations, and ensured that workshop processes reflected local priorities. The multisectoral workshops were co-facilitated with Filipino colleagues to reduce power imbalances and promote open discussion. Throughout data analysis, the researcher sought regular feedback from local collaborators to check assumptions and ensure that interpretations remained grounded in local realities.

The author acknowledges that, despite these measures, her positionality inevitably shaped the research process. Making this positionality explicit is intended to increase transparency and support a more nuanced understanding of how knowledge was co-produced within this study.
